# Supplementary material for: Less need for differentiation? Intestinal length of reptiles as compared to mammals
Source: PLoS One. 2021 Jul 2;16(7):e0253182. doi: 10.1371/journal.pone.0253182 (PMC8253402; doi:10.1371/journal.pone.0253182)
Supplement: S1 File — S1-S8 Tables & S1 Fig. (DOCX) [file pone.0253182.s001.docx]

**S1 File**

**Less need for differentiation? Intestinal morphology of reptiles as compared to mammals**

Monika I. Hoppe, Carlo Meloro, Mark S. Edwards, Daryl Codron, Marcus Clauss, María J. Duque-Correa

S1-S8 Tables

S1 Figure

**S1 Table** Summary statistics for allometric scaling as log(y) = a + b log(body mass), or y = (10^a^) BM^b^ (significant parameters in **bold**)

| **Dependent** |  |  | **GLS parameter** | **PGLS** | **parameter** |
| --- | --- | --- | --- | --- | --- |
|  | **n** |  | **(95% CI)** | **lambda** | **(95% CI)** |
|  |  |  |  |  |  |
| *Squamates (lizards and snakes)* | | | | | |
| Total intest. | 137 | a | **1.79 (1.75 to 1.84)** | **0.55 (0.28 to 0.79)** | **1.72 (1.63 to 1.81)** |
|  |  | b | **0.38 (0.35 to 0.41)** |  | **0.36 (0.33 to 0.39)** |
| Small intest. | 127 | a | **1.67 (1.61 to 1.72)** | **0.31 (0.13 to 0.56)** | **1.58 (1.50 to 1.67)** |
|  |  | b | **0.39 (0.35 to 0.42)** |  | **0.36 (0.33 to 0.40)** |
| Large intest. | 122 | a | **1.21 (1.15 to 1.27)** | **0.13 (0.01 to 0.39)** | **1.17 (1.09 to 1.25)** |
|  |  | b | **0.41 (0.37 to 0.44)** |  | **0.39 (0.36 to 0.43)** |
| Caecum | 46 | a | **0.46 (0.34 to 0.59)** | 0.00 (NA to 0.24) | **0.46 (0.34 to 0.59)** |
|  |  | b | **0.40 (0.33 to 0.48)** |  | **0.40 (0.33 to 0.48)** |
|  |  |  |  |  |  |
| *Lizards* | | | | | |
| Total intest. | 84 | a | **1.78 (1.72 to 1.84)** | **0.51 (0.16 to 0.83)** | **1.75 (1.66 to 1.83)** |
|  |  | b | **0.41 (0.37 to 0.44)** |  | **0.39 (0.36 to 0.43)** |
| Small intest. | 71 | a | **1.60 (1.54 to 1.66)** | **0.33 (0.06 to 0.69)** | **1.59 (1.51 to 1.66)** |
|  |  | b | **0.39 (0.36 to 0.42)** |  | **0.39 (0.35 to 0.42)** |
| Large intest. | 69 | a | **1.23 (1.16 to 1.30)** | 0.17 (NA to 0.61) | **1.22 (1.13 to 1.31)** |
|  |  | b | **0.45 (0.40 to 0.49)** |  | **0.44 (0.39 to 0.49)** |
| Caecum | 24 | a | **0.54 (0.34 to 0.74)** | 0.00 (NA to 0.34) | **0.54 (0.34 to 0.74)** |
|  |  | b | **0.43 (0.30 to 0.57)** |  | **0.43 (0.30 to 0.57)** |
|  |  |  |  |  |  |
| *Snakes* | | | | | |
| Total intest. | 53 | a | **1.81 (1.75 to 1.87)** | 0.34 (NA to 0.83) | **1.83 (1.72 to 1.94)** |
|  |  | b | **0.33 (0.29 to 0.37)** |  | **0.34 (0.30 to 0.38)** |
| Small intest. | 56 | a | **1.72 (1.64 to 1.79)** | 0.00 (NA to 0.41) | **1.72 (1.64 to 1.79)** |
|  |  | b | **0.35 (0.30 to 0.40)** |  | **0.35 (0.30 to 0.40)** |
| Large intest. | 53 | a | **1.20 (1.12 to 1.27)** | 0.00 (NA to 0.19) | **1.20 (1.12 to 1.27)** |
|  |  | b | **0.35 (0.30 to 0.40)** |  | **0.35 (0.30 to 0.40)** |
| Caecum | 22 | a | **0.40 (0.23 to 0.57)** | 0.00 (NA to 0.51) | **0.40 (0.23 to 0.57)** |
|  |  | b | **0.39 (0.30 to 0.48)** |  | **0.39 (0.30 to 0.48)** |
|  |  |  |  |  |  |
| *Turtles* | | | | | |
| Total intest. | 18 | a | **1.79 (1.69 to 1.89)** | **0.90 (0.19 to NA)** | **1.77 (1.53 to 2.01)** |
|  |  | b | **0.52 (0.42 to 0.62)** |  | **0.45 (0.36 to 0.55)** |
| Small intest. | 18 | a | **1.63 (1.52 to 1.73)** | **1.00 (0.66 to NA)** | **1.64 (1.34 to 1.93)** |
|  |  | b | **0.40 (0.28 to 0.52)** |  | **0.34 (0.25 to 0.44)** |
| Large intest. | 17 | a | **1.26 (1.11 to 1.40)** | **0.85 (0.26 to NA)** | **1.21 (0.91 to 1.51)** |
|  |  | b | **0.60 (0.43 to 0.76)** |  | **0.55 (0.42 to 0.68)** |
| Caecum | 6 | a | **0.43 (0.19 to 0.67)** | 0.00 (NA to NA) | **0.43 (0.19 to 0.67)** |
|  |  | b | **0.70 (0.46 to 0.94)** |  | **0.70 (0.46 to 0.94)** |

NA no model output

**S2 Table** Summary statistics for allometric scaling as log(y) = a + b log(body mass) + c (taxon), or y = (10^a^) (10^c^) BM^b^ (significant parameters in **bold**); data for mammals (Duque-Correa et al. 2021) compared to the reptile compilation of the present study

| **Dependent** | **Model** |  |  | **GLS** |  | **parameter** | **PGLS** |  |  | **parameter** |
| --- | --- | --- | --- | --- | --- | --- | --- | --- | --- | --- |
|  |  | **n** |  | **AICc** | **ΔAIC** | **(95% CI)** | **lambda** | **AICc** | **ΔAIC** | **(95% CI)** |
| Total intest. | BM | 676 | a | 124.4 | 253.7 | **2.19 (2.17 to 2.21)** | **0.92 (0.88 to 0.95)** | -609.1 | 0.0 | **1.91 (1.61 to 2.21)** |
|  |  |  | b |  |  | **0.50 (0.49 to 0.51)** |  |  |  | **0.40 (0.38 to 0.42)** |
|  | BM + taxon | 676 | a | -119.6 | 9.7 | **2.26 (2.24 to 2.28)** | **0.92 (0.88 to 0.95)** | -608.7 | 0.5 | **2.17 (1.66 to 2.68)** |
|  |  |  | b |  |  | **0.46 (0.45 to 0.48)** |  |  |  | **0.4 (0.38 to 0.42)** |
|  |  |  | cRept |  |  | **-0.37 (-0.41 to -0.32)** |  |  |  | -0.40 (-1.02 to 0.23) |
|  | BM x taxon | 676 | a | -129.3 | 0.0 | **2.26 (2.24 to 2.28)** | **0.92 (0.88 to 0.94)** | -608.9 | 0.2 | **2.17 (1.67 to 2.68)** |
|  |  |  | b |  |  | **0.48 (0.46 to 0.49)** |  |  |  | **0.41 (0.39 to 0.43)** |
|  |  |  | cRept |  |  | **-0.44 (-0.49 to -0.39)** |  |  |  | -0.41 (-1.03 to 0.21) |
|  |  |  | b x c |  |  | **-0.07 (-0.11 to -0.04)** |  |  |  | -0.03 (-0.08 to 0.01) |
|  |  |  |  |  |  |  |  |  |  |  |
| Small intest. | BM | 544 | a | 113.4 | 264.0 | **2.03 (2.01 to 2.05)** | **0.91 (0.86 to 0.94)** | -415.4 | 0.5 | **1.77 (1.47 to 2.08)** |
|  |  |  | b |  |  | **0.50 (0.49 to 0.52)** |  |  |  | **0.39 (0.37 to 0.41)** |
|  | BM + taxon | 544 | a | -140.5 | 10.1 | **2.14 (2.12 to 2.16)** | **0.91 (0.85 to 0.94)** | -415.0 | 0.9 | **2.04 (1.53 to 2.56)** |
|  |  |  | b |  |  | **0.44 (0.43 to 0.46)** |  |  |  | **0.39 (0.37 to 0.41)** |
|  |  |  | cRept |  |  | **-0.41 (-0.45 to -0.37)** |  |  |  | -0.41 (-1.05 to 0.22) |
|  | BM x taxon | 544 | a | -150.7 | 0.0 | **2.13 (2.11 to 2.15)** | **0.90 (0.85 to 0.94)** | -416.0 | 0.0 | **2.04 (1.53 to 2.56)** |
|  |  |  | b |  |  | **0.46 (0.44 to 0.47)** |  |  |  | **0.40 (0.38 to 0.43)** |
|  |  |  | cRept |  |  | **-0.47 (-0.53 to -0.42)** |  |  |  | -0.43 (-1.06 to 0.21) |
|  |  |  | b x c |  |  | **-0.08 (-0.11 to -0.04)** |  |  |  | -0.04 (-0.09 to 0.01) |
|  |  |  |  |  |  |  |  |  |  |  |
| Large intest. | BM | 528 | a | 462.9 | 187.6 | **1.61 (1.58 to 1.65)** | **0.95 (0.93 to 0.97)** | -218.9 | 0.0 | **1.27 (0.83 to 1.71)** |
|  |  |  | b |  |  | **0.49 (0.47 to 0.51)** |  |  |  | **0.38 (0.35 to 0.41)** |
|  | BM + taxon | 528 | a | 275.4 | 0.0 | **1.75 (1.72 to 1.78)** | **0.95 (0.92 to 0.97)** | -217.9 | 1.0 | **1.57 (0.83 to 2.32)** |
|  |  |  | b |  |  | **0.41 (0.39 to 0.44)** |  |  |  | **0.38 (0.35 to 0.41)** |
|  |  |  | cRept |  |  | **-0.52 (-0.59 to -0.45)** |  |  |  | -0.47 (-1.39 to 0.46) |
|  | BM x taxon | 528 | a | 282.3 | 7.0 | **1.75 (1.72 to 1.78)** | **0.95 (0.92 to 0.97)** | -216.0 | 3.0 | **1.57 (0.82 to 2.32)** |
|  |  |  | b |  |  | **0.41 (0.39 to 0.43)** |  |  |  | **0.38 (0.35 to 0.41)** |
|  |  |  | cRept |  |  | **-0.50 (-0.58 to -0.42)** |  |  |  | -0.47 (-1.39 to 0.46) |
|  |  |  | b x c |  |  | 0.02 (-0.03 to 0.07) |  |  |  | -0.01 (-0.07 to 0.06) |
|  |  |  |  |  |  |  |  |  |  |  |
| Caecum | BM | 404 | a | 436.7 | 157.1 | **0.88 (0.84 to 0.92)** | **0.98 (0.97 to 0.99)** | -117.0 | 0.1 | 0.54 (-0.08 to 1.16) |
|  |  |  | b |  |  | **0.30 (0.27 to 0.33)** |  |  |  | **0.30 (0.26 to 0.33)** |
|  | BM + taxon | 404 | a | 296.6 | 17.0 | **0.98 (0.94 to 1.01)** | **0.98 (0.97 to 0.99)** | -115.1 | 1.9 | 0.71 (-0.28 to 1.70) |
|  |  |  | b |  |  | **0.23 (0.21 to 0.26)** |  |  |  | **0.30 (0.26 to 0.33)** |
|  |  |  | cRept |  |  | **-0.72 (-0.83 to -0.61)** |  |  |  | -0.28 (-1.55 to 0.99) |
|  | BM x taxon | 404 | a | 279.6 | 0.0 | **0.98 (0.95 to 1.02)** | **0.98 (0.96 to 0.99)** | -117.0 | 0.0 | 0.72 (-0.26 to 1.69) |
|  |  |  | b |  |  | **0.21 (0.18 to 0.24)** |  |  |  | **0.28 (0.25 to 0.32)** |
|  |  |  | cRept |  |  | **-0.50 (-0.64 to -0.37)** |  |  |  | -0.22 (-1.46 to 1.03) |
|  |  |  | b x c |  |  | **0.21 (0.13 to 0.30)** |  |  |  | **0.10 (0.00 to 0.21)** |

**S3 Table** Summary statistics for models assessing different reptilian intestinal sections only with body mass (BM) or additionally with a diet proxy according to log(length) = a + b log(BM) + c (trophic level) , or y = (10^a^) (10^c^) BM^b^; (significant parameters in **bold**)

| **Dependent** | **Model** |  |  | **GLS** |  | **parameter** | **PGLS** |  |  | **parameter** |
| --- | --- | --- | --- | --- | --- | --- | --- | --- | --- | --- |
|  |  | **n** |  | **AICc** | **ΔAIC** | **(95%CI)** | **lambda (95%CI)** | **AICc** | **ΔAIC** | **(95% CI)** |
| Total intest. | BM | 157 | a | -116.3 | 0.0 | **1.82 (1.79 to 1.86)** | **0.77 (0.56 to 0.90)** | -177.1 | 0.2 | **1.77 (1.61 to 1.92)** |
|  |  |  | b |  |  | **0.40 (0.38 to 0.42)** |  |  |  | **0.38 (0.35 to 0.40)** |
| Total intest. | BM + trophic | 157 | a | -108.6 | 7.7 | **1.83 (1.78 to 1.87)** | **0.78 (0.58 to 0.90)** | -177.3 | 0.0 | **1.74 (1.58 to 1.90)** |
|  |  |  | b |  |  | **0.40 (0.37 to 0.42)** |  |  |  | **0.37 (0.34 to 0.40)** |
|  |  |  | cHerb |  |  | 0.03 (-0.05 to 0.11) |  |  |  | 0.09 (0.00 to 0.18) |
|  |  |  | cOmni |  |  | **-0.07 (-0.13 to -0.01)** |  |  |  | 0.01 (-0.05 to 0.07) |
|  |  |  |  |  |  |  |  |  |  |  |
| Small intest. | BM | 147 | a | -74.5 | 0.0 | **1.66 (1.62 to 1.70)** | **0.50 (0.22 to 0.74)** | -114.3 | 0.0 | **1.62 (1.49 to 1.75)** |
|  |  |  | b |  |  | **0.38 (0.36 to 0.41)** |  |  |  | **0.37 (0.33 to 0.40)** |
| Small intest. | BM + trophic | 147 | a | -64.7 | 9.8 | **1.68 (1.63 to 1.73)** | **0.51 (0.23 to 0.75)** | -110.6 | 3.7 | **1.61 (1.47 to 1.74)** |
|  |  |  | b |  |  | **0.39 (0.36 to 0.42)** |  |  |  | **0.36 (0.33 to 0.40)** |
|  |  |  | cHerb |  |  | -0.05 (-0.15 to 0.04) |  |  |  | 0.02 (-0.09 to 0.13) |
|  |  |  | cOmni |  |  | -0.06 (-0.13 to 0.02) |  |  |  | 0.02 (-0.06 to 0.10) |
|  |  |  |  |  |  |  |  |  |  |  |
| Large intest. | BM | 141 | a | -26.5 | 0.0 | **1.24 (1.19 to 1.29)** | **0.56 (0.31 to 0.75)** | -61.7 | 2.4 | **1.14 (0.98 to 1.31)** |
|  |  |  | b |  |  | **0.43 (0.40 to 0.46)** |  |  |  | **0.40 (0.37 to 0.44)** |
| Large intest. | BM + trophic | 141 | a | -21.5 | 5.0 | **1.22 (1.16 to 1.28)** | **0.56 (0.30 to 0.75)** | -64.1 | 0.0 | **1.10 (0.93 to 1.27)** |
|  |  |  | b |  |  | **0.41 (0.38 to 0.45)** |  |  |  | **0.39 (0.35 to 0.43)** |
|  |  |  | cHerb |  |  | **0.12 (0.02 to 0.23)** |  |  |  | **0.16 (0.03 to 0.28)** |
|  |  |  | cOmni |  |  | -0.05 (-0.14 to 0.04) |  |  |  | 0.01 (-0.08 to 0.11) |
|  |  |  |  |  |  |  |  |  |  |  |
| Caecum | BM | 52 | a | 29.8 | 0.0 | **0.48 (0.37 to 0.59)** | 0.00 (NA to 0.27) | 18.3 | 0.0 | **0.48 (0.37 to 0.59)** |
|  |  |  | b |  |  | **0.42 (0.35 to 0.49)** |  |  |  | **0.42 (0.35 to 0.49)** |
| Caecum | BM + trophic | 52 | a | 37.9 | 8.1 | **0.44 (0.29 to 0.59)** | 0.00 (NA to 0.30) | 21.4 | 3.1 | **0.44 (0.29 to 0.59)** |
|  |  |  | b |  |  | **0.41 (0.33 to 0.49)** |  |  |  | **0.41 (0.33 to 0.49)** |
|  |  |  | cHerb |  |  | 0.08 (-0.13 to 0.30) |  |  |  | 0.08 (-0.13 to 0.30) |
|  |  |  | cOmni |  |  | 0.09 (-0.16 to 0.34) |  |  |  | 0.09 (-0.16 to 0.34) |

NA no model output

***S3 Table ctd.*** Summary statistics for models assessing different reptilian intestinal sections only with body mass (BM) or additionally with a diet proxy according to log(length) = a + b log(BM) + c (trophic level) , or y = (10^a^) (10^c^) BM^b^; (significant parameters in **bold**)

|  |  |  |  | **GLS** |  | |  | **parameter** | **PGLS** |  |  |  | **parameter** |
| --- | --- | --- | --- | --- | --- | --- | --- | --- | --- | --- | --- | --- | --- |
| **Dependent** | **Model** | **n** |  | **AICc** | **ΔAIC (trophic)** | | **ΔAIC (all)** | **(95%CI)** | **lambda (95%CI)** | **AICc** | **ΔAIC (trophic)** | **ΔAIC (all)** | **(95% CI)** |
|  | | | | | | | | | | | | | |
| *Consistent data (species for which both small and large intestinal length are available)* | | | | | | | | | | | | | |
| Small intest. | BM | 141 | a | -82.3 | 0.0 | 0.0 | | **1.64 (1.60 to 1.68)** | **0.64 (0.34 to 0.84)** | -125.5 | 0.0 | 0.0 | **1.61 (1.46 to 1.75)** |
|  |  |  | b |  |  |  | | **0.38 (0.35 to 0.40)** |  |  |  |  | **0.36 (0.33 to 0.39)** |
| Small intest. | BM + trophic | 141 | a | -71.8 | 10.5 | 10.5 | | **1.66 (1.61 to 1.71)** | **0.65 (0.36 to 0.84)** | -122.2 | 3.3 | 3.3 | **1.59 (1.44 to 1.75)** |
|  |  |  | b |  |  |  | | **0.38 (0.35 to 0.41)** |  |  |  |  | **0.36 (0.33 to 0.39)** |
|  |  |  | cHerb |  |  |  | | -0.04 (-0.13 to 0.06) |  |  |  |  | 0.04 (-0.07 to 0.14) |
|  |  |  | cOmni |  |  |  | | -0.06 (-0.13 to 0.02) |  |  |  |  | 0.02 (-0.05 to 0.10) |
|  |  |  |  |  |  |  | |  |  |  |  |  |  |
| Large intest. | BM | 141 | a | -26.5 | 0.0 | 55.8 | | **1.24 (1.19 to 1.29)** | **0.56 (0.31 to 0.75)** | -61.7 | 2.4 | 63.8 | **1.14 (0.98 to 1.31)** |
|  |  |  | b |  |  |  | | **0.43 (0.40 to 0.46)** |  |  |  |  | **0.40 (0.37 to 0.44)** |
| Large intest. | BM + trophic | 141 | a | -21.5 | 5.0 | 60.8 | | **1.22 (1.16 to 1.28)** | **0.56 (0.30 to 0.75)** | -64.1 | 0.0 | 61.4 | **1.10 (0.93 to 1.27)** |
|  |  |  | b |  |  |  | | **0.41 (0.38 to 0.45)** |  |  |  |  | **0.39 (0.35 to 0.43)** |
|  |  |  | cHerb |  |  |  | | **0.12 (0.02 to 0.23)** |  |  |  |  | **0.16 (0.03 to 0.28)** |
|  |  |  | cOmni |  |  |  | | -0.05 (-0.14 to 0.04) |  |  |  |  | 0.01 (-0.08 to 0.11) |

**S4 Table** Summary statistics for models assessing different reptilian intestinal sections only with body mass (BM) or additionally with a diet proxy according to log(length) = a + b log(BM) + c (trophic level) , or y = (10^a^) (10^c^) BM^b^; (significant parameters in **bold**)

| **Dependent** |  |  |  | **GLS** |  | **parameter** | **PGLS** |  |  | **parameter** |
| --- | --- | --- | --- | --- | --- | --- | --- | --- | --- | --- |
|  | **Model** | **n** |  | **AICc** | **ΔAIC** | **(95% CI)** | **lambda** | **AICc** | **ΔAIC** | **(95% CI)** |
| *Squamates* |  |  |  |  |  |  |  |  |  |  |
| Total intest. | BM | 137 | a | -116.5 | 0.0 | **1.79 (1.75 to 1.84)** | **0.55 (0.28 to 0.79)** | -169.6 | 6.1 | **1.72 (1.63 to 1.81)** |
|  |  |  | b |  |  | **0.38 (0.35 to 0.41)** |  |  |  | **0.36 (0.33 to 0.39)** |
|  | BM + trophic | 137 | a | -112.9 | 3.6 | **1.78 (1.73 to 1.83)** | **0.50 (0.25 to 0.75)** | -175.7 | 0.0 | **1.68 (1.60 to 1.77)** |
|  |  |  | b |  |  | **0.37 (0.34 to 0.40)** |  |  |  | **0.35 (0.32 to 0.38)** |
|  |  |  | cHerb |  |  | 0.09 (0.00 to 0.19) |  |  |  | **0.17 (0.06 to 0.27)** |
|  |  |  | cOmni |  |  | **-0.07 (-0.13 to -0.01)** |  |  |  | 0.01 (-0.05 to 0.07) |
|  |  |  |  |  |  |  |  |  |  |  |
| SI | BM | 127 | a | -68.5 | 0.0 | **1.67 (1.61 to 1.72)** | **0.31 (0.13 to 0.56)** | -108.6 | 0.0 | **1.58 (1.50 to 1.67)** |
|  |  |  | b |  |  | **0.39 (0.35 to 0.42)** |  |  |  | **0.36 (0.33 to 0.40)** |
|  | BM + trophic | 127 | a | -59.7 | 8.8 | **1.67 (1.62 to 1.73)** | **0.32 (0.14 to 0.56)** | -107.7 | 0.9 | **1.56 (1.46 to 1.65)** |
|  |  |  | b |  |  | **0.38 (0.35 to 0.41)** |  |  |  | **0.35 (0.32 to 0.39)** |
|  |  |  | cHerb |  |  | 0.02 (-0.09 to 0.13) |  |  |  | 0.11 (-0.01 to 0.24) |
|  |  |  | cOmni |  |  | -0.08 (-0.16 to 0.01) |  |  |  | 0.01 (-0.07 to 0.10) |
|  |  |  |  |  |  |  |  |  |  |  |
| LI | BM | 122 | a | -59.9 | 0.0 | **1.21 (1.15 to 1.27)** | **0.13 (0.01 to 0.39)** | -79.1 | 3.9 | **1.17 (1.09 to 1.25)** |
|  |  |  | b |  |  | **0.41 (0.37 to 0.44)** |  |  |  | **0.39 (0.36 to 0.43)** |
|  | BM + trophic | 122 | a | -53.9 | 6.0 | **1.19 (1.13 to 1.25)** | **0.15 (0.02 to 0.38)** | -83.0 | 0.0 | **1.12 (1.04 to 1.21)** |
|  |  |  | b |  |  | **0.39 (0.36 to 0.43)** |  |  |  | **0.37 (0.34 to 0.41)** |
|  |  |  | cHerb |  |  | **0.13 (0.01 to 0.25)** |  |  |  | **0.19 (0.05 to 0.32)** |
|  |  |  | cOmni |  |  | -0.05 (-0.14 to 0.03) |  |  |  | 0.00 (-0.09 to 0.09) |
|  |  |  |  |  |  |  |  |  |  |  |
| Cec | BM | 46 | a | 22.5 | 0.0 | **0.46 (0.34 to 0.59)** | 0.00 (NA to 0.24) | 11.1 | 0.0 | **0.46 (0.34 to 0.59)** |
|  |  |  | b |  |  | **0.40 (0.33 to 0.48)** |  |  |  | **0.40 (0.33 to 0.48)** |
|  | BM + trophic | 46 | a | 28.0 | 5.5 | **0.40 (0.25 to 0.54)** | 0.00 (NA to 0.21) | 11.2 | 0.1 | **0.40 (0.25 to 0.54)** |
|  |  |  | b |  |  | **0.38 (0.31 to 0.46)** |  |  |  | **0.38 (0.31 to 0.46)** |
|  |  |  | cHerb |  |  | 0.18 (-0.06 to 0.42) |  |  |  | 0.18 (-0.06 to 0.42) |
|  |  |  | cOmni |  |  | 0.15 (-0.10 to 0.40) |  |  |  | 0.15 (-0.10 to 0.40) |
|  |  |  |  |  |  |  |  |  |  |  |
| *Lizards* |  |  |  |  |  |  |  |  |  |  |
| Total intest. | BM | 84 | a | -97.0 | 2.9 | **1.78 (1.72 to 1.84)** | **0.51 (0.16 to 0.83)** | -124.9 | 8.3 | **1.75 (1.66 to 1.83)** |
|  |  |  | b |  |  | **0.41 (0.37 to 0.44)** |  |  |  | **0.39 (0.36 to 0.43)** |
|  | BM + trophic | 84 | a | -99.9 | 0.0 | **1.69 (1.62 to 1.76)** | **0.35 (0.07 to 0.73)** | -133.2 | 0.0 | **1.68 (1.59 to 1.76)** |
|  |  |  | b |  |  | **0.36 (0.33 to 0.40)** |  |  |  | **0.36 (0.32 to 0.40)** |
|  |  |  | cHerb |  |  | **0.18 (0.10 to 0.27)** |  |  |  | **0.17 (0.08 to 0.26)** |
|  |  |  | cOmni |  |  | 0.01 (-0.04 to 0.07) |  |  |  | 0.02 (-0.03 to 0.07) |
|  |  |  |  |  |  |  |  |  |  |  |
| SI | BM | 71 | a | -76.1 | 0.0 | **1.60 (1.54 to 1.66)** | **0.33 (0.06 to 0.69)** | -98.0 | 2.8 | **1.59 (1.51 to 1.66)** |
|  |  |  | b |  |  | **0.39 (0.36 to 0.42)** |  |  |  | **0.39 (0.35 to 0.42)** |
|  | BM + trophic | 71 | a | -73.9 | 2.2 | **1.52 (1.44 to 1.59)** | 0.19 (NA to 0.59) | -100.8 | 0.0 | **1.52 (1.44 to 1.60)** |
|  |  |  | b |  |  | **0.36 (0.32 to 0.39)** |  |  |  | **0.36 (0.32 to 0.40)** |
|  |  |  | cHerb |  |  | **0.16 (0.07 to 0.25)** |  |  |  | **0.14 (0.04 to 0.24)** |
|  |  |  | cOmni |  |  | 0.04 (-0.03 to 0.10) |  |  |  | 0.03 (-0.03 to 0.10) |
|  |  |  |  |  |  |  |  |  |  |  |
| LI | BM | 69 | a | -46.8 | 0.0 | **1.23 (1.16 to 1.30)** | 0.17 (NA to 0.61) | -62.0 | 3.8 | **1.22 (1.13 to 1.31)** |
|  |  |  | b |  |  | **0.45 (0.40 to 0.49)** |  |  |  | **0.44 (0.39 to 0.49)** |
|  | BM + trophic | 69 | a | -43.0 | 3.8 | **1.13 (1.04 to 1.23)** | 0.04 (NA to 0.48) | -65.8 | 0.0 | **1.13 (1.03 to 1.23)** |
|  |  |  | b |  |  | **0.40 (0.35 to 0.45)** |  |  |  | **0.40 (0.35 to 0.45)** |
|  |  |  | cHerb |  |  | **0.18 (0.06 to 0.31)** |  |  |  | **0.18 (0.06 to 0.31)** |
|  |  |  | cOmni |  |  | 0.02 (-0.06 to 0.10) |  |  |  | 0.02 (-0.05 to 0.10) |
|  |  |  |  |  |  |  |  |  |  |  |
| Cec | BM | 24 | a | 15.7 | 0.0 | **0.54 (0.34 to 0.74)** | 0.00 (NA to 0.34) | 6.1 | 0.0 | **0.54 (0.34 to 0.74)** |
|  |  |  | b |  |  | **0.43 (0.30 to 0.57)** |  |  |  | **0.43 (0.30 to 0.57)** |
|  | BM + trophic | 24 | a | 21.8 | 6.1 | **0.38 (0.07 to 0.69)** | 0.00 (NA to 0.38) | 7.9 | 1.8 | **0.38 (0.07 to 0.69)** |
|  |  |  | b |  |  | **0.37 (0.20 to 0.53)** |  |  |  | **0.37 (0.20 to 0.53)** |
|  |  |  | cHerb |  |  | 0.20 (-0.12 to 0.52) |  |  |  | 0.20 (-0.12 to 0.52) |
|  |  |  | cOmni |  |  | 0.16 (-0.13 to 0.44) |  |  |  | 0.16 (-0.13 to 0.44) |

***S4 Table ctd.*** Summary statistics for models assessing different reptilian intestinal sections only with body mass (BM) or additionally with a diet proxy according to log(length) = a + b log(BM) + c (trophic level) , or y = (10^a^) (10^c^) BM^b^; (significant parameters in **bold**)

| **Dependent** |  |  |  | **GLS** |  | **parameter** | **PGLS** |  |  | **parameter** |
| --- | --- | --- | --- | --- | --- | --- | --- | --- | --- | --- |
|  | **Model** | **n** |  | **AICc** | **ΔAIC** | **(95% CI)** | **lambda** | **AICc** | **ΔAIC** | **(95% CI)** |
| *Turtles* |  |  |  |  |  |  |  |  |  |  |
| Total intest. | BM | 18 | a | 3.8 | 0.0 | **1.79 (1.69 to 1.89)** | **0.90 (0.19 to NA)** | -13.0 | 0.0 | **1.77 (1.53 to 2.01)** |
|  |  |  | b |  |  | **0.52 (0.42 to 0.62)** |  |  |  | **0.45 (0.36 to 0.55)** |
|  | BM + trophic | 18 | a | 10.9 | 7.1 | **1.96 (1.67 to 2.24)** | **0.96 (0.06 to NA)** | -9.3 | 3.7 | **1.71 (1.36 to 2.07)** |
|  |  |  | b |  |  | **0.48 (0.36 to 0.60)** |  |  |  | **0.45 (0.35 to 0.55)** |
|  |  |  | cHerb |  |  | -0.17 (-0.46 to 0.12) |  |  |  | 0.06 (-0.17 to 0.30) |
|  |  |  | cOmni |  |  | -0.20 (-0.51 to 0.11) |  |  |  | 0.06 (-0.21 to 0.33) |
|  |  |  |  |  |  |  |  |  |  |  |
| SI | BM | 18 | a | 7.7 | 0.0 | **1.63 (1.52 to 1.73)** | **1.00 (0.66 to NA)** | -10.5 | 0.0 | **1.64 (1.34 to 1.93)** |
|  |  |  | b |  |  | **0.40 (0.28 to 0.52)** |  |  |  | **0.34 (0.25 to 0.44)** |
|  | BM + trophic | 18 | a | 12.9 | 5.2 | **1.83 (1.50 to 2.15)** | **1.00 (0.48 to NA)** | -7.3 | 3.2 | **1.67 (1.27 to 2.07)** |
|  |  |  | b |  |  | **0.38 (0.25 to 0.51)** |  |  |  | **0.35 (0.25 to 0.46)** |
|  |  |  | cHerb |  |  | -0.27 (-0.61 to 0.07) |  |  |  | -0.17 (-0.35 to 0.21) |
|  |  |  | cOmni |  |  | -0.15 (-0.50 to 0.21) |  |  |  | -0.02 (-0.32 to 0.28) |
|  |  |  |  |  |  |  |  |  |  |  |
| LI | BM | 17 | a | 15.3 | 0.0 | **1.26 (1.11 to 1.40)** | **0.85 (0.26 to NA)** | -2.6 | 0.0 | **1.21 (0.91 to 1.51)** |
|  |  |  | b |  |  | **0.60 (0.43 to 0.76)** |  |  |  | **0.55 (0.42 to 0.68)** |
|  | BM + trophic | 17 | a | 19.2 | 3.9 | **1.51 (1.09 to 1.93)** | **0.92 (0.14 to NA)** | 0.5 | 3.1 | **1.10 (0.63 to 1.57)** |
|  |  |  | b |  |  | **0.55 (0.38 to 0.72)** |  |  |  | **0.53 (0.39 to 0.67)** |
|  |  |  | cHerb |  |  | -0.19 (-0.62 to 0.24) |  |  |  | 0.15 (-0.20 to 0.50) |
|  |  |  | cOmni |  |  | -0.39 (-0.84 to 0.07) |  |  |  | 0.10 (-0.28 to 0.48) |
|  |  |  |  |  |  |  |  |  |  |  |
| Cec | BM | 6 | a | 11.3 | 0.0 | **0.43 (0.19 to 0.67)** | 0.00 (NA to NA) | 4.1 | 0.0 | **0.43 (0.19 to 0.67)** |
|  |  |  | b |  |  | **0.70 (0.46 to 0.94)** |  |  |  | **0.70 (0.46 to 0.94)** |
|  | BM + trophic | 6 | a | 13.0 | 1.8 | **0.40 (0.10 to 0.69)** | 0.00 (NA to NA) | 5.6 | 1.5 | **0.40 (0.10 to 0.69)** |
|  |  |  | b |  |  | **0.74 (0.43 to 1.04)** |  |  |  | **0.74 (0.43 to 1.04)** |
|  |  |  | cHerb |  |  | - |  |  |  | - |
|  |  |  | cOmni |  |  | 0.21 (-0.60 to 1.01) |  |  |  | 0.21 (-0.60 to 1.01) |

NA no model output

**S5 Table** Summary statistics for allometric scaling as log(y) = a + b log(body mass) + c (taxon) + d (trophic level), or y = (10^a^) (10^c^) (10^d^) BM^b^ (significant parameters in **bold**); data for mammals (Duque-Correa et al. 2021) compared to the reptile compilation of the present study

| **Dependent** | **Model** |  |  | **GLS** |  | **parameter** | **PGLS** |  |  | **parameter** |
| --- | --- | --- | --- | --- | --- | --- | --- | --- | --- | --- |
|  |  | **n** |  | **AICc** | **ΔAIC** | **(95% CI)** | **lambda** | **AICc** | **ΔAIC** | **(95% CI)** |
| Total intest. | BM | 676 | a | 124.4 | 343.2 | **2.19 (2.17 to 2.21)** | **0.92 (0.88 to 0.95)** | -609.1 | 5.3 | **1.91 (1.61 to 2.21)** |
|  |  |  | b |  |  | **0.50 (0.49 to 0.51)** |  |  |  | **0.40 (0.38 to 0.42)** |
|  | BM + trophic | 676 | a | -18.3 | 200.5 | **2.02 (1.99 to 2.05)** | **0.91 (0.87 to 0.94)** | -614.4 | 0.0 | **1.88 (1.60 to 2.17)** |
|  |  |  | b |  |  | **0.46 (0.45 to 0.47)** |  |  |  | **0.39 (0.38 to 0.41)** |
|  |  |  | dHerb |  |  | **0.30 (0.26 to 0.35)** |  |  |  | **0.08 (0.03 to 0.14)** |
|  |  |  | dOmni |  |  | **0.14 (0.10 to 0.19)** |  |  |  | **0.04 (-0.01 to 0.08)** |
|  | BM + taxon | 676 | a | -119.6 | 99.2 | **2.26 (2.24 to 2.28)** | **0.92 (0.88 to 0.95)** | -608.7 | 5.7 | **2.17 (1.66 to 2.68)** |
|  |  |  | b |  |  | **0.46 (0.45 to 0.48)** |  |  |  | **0.40 (0.38 to 0.42)** |
|  |  |  | cRept |  |  | **-0.37 (-0.41 to -0.32)** |  |  |  | -0.40 (-1.02 to 0.23) |
|  | BM + taxon + trophic | 676 | a | -218.8 | 0.0 | **2.13 (2.10 to 2.16)** | **0.91 (0.86 to 0.94)** | -614.0 | 0.4 | **2.14 (1.66 to 2.63)** |
|  |  |  | b |  |  | **0.44 (0.43 to 0.45)** |  |  |  | **0.39 (0.38 to 0.41)** |
|  |  |  | cRept |  |  | **-0.31 (-0.35 to -0.27)** |  |  |  | -0.39 (-0.99 to 0.20) |
|  |  |  | dHerb |  |  | **0.22 (0.18 to 0.26)** |  |  |  | **0.08 (0.03 to 0.14)** |
|  |  |  | dOmni |  |  | **0.08 (0.04 to 0.12)** |  |  |  | 0.04 (-0.01 to 0.08) |
|  |  |  |  |  |  |  |  |  |  |  |
| Small intest. | BM | 544 | a | 113.4 | 253.9 | **2.03 (2.01 to 2.05)** | **0.91 (0.86 to 0.94)** | -415.4 | 0.0 | **1.77 (1.47 to 2.08)** |
|  |  |  | b |  |  | **0.50 (0.49 to 0.52)** |  |  |  | **0.39 (0.37 to 0.41)** |
|  | BM + trophic | 544 | a | 59.6 | 200.1 | **1.89 (1.85 to 1.93)** | **0.91 (0.86 to 0.94)** | -412.4 | 3.0 | **1.76 (1.45 to 2.07)** |
|  |  |  | b |  |  | **0.48 (0.46 to 0.50)** |  |  |  | **0.39 (0.37 to 0.42)** |
|  |  |  | dHerb |  |  | **0.23 (0.17 to 0.28)** |  |  |  | 0.02 (-0.05 to 0.09) |
|  |  |  | dOmni |  |  | **0.16 (0.11 to 0.22)** |  |  |  | 0.03 (-0.03 to 0.09) |
|  | BM + taxon | 544 | a | -140.5 | 0.0 | **2.14 (2.12 to 2.16)** | **0.91 (0.85 to 0.94)** | -415.0 | 0.4 | **2.04 (1.53 to 2.56)** |
|  |  |  | b |  |  | **0.44 (0.43 to 0.46)** |  |  |  | **0.39 (0.37 to 0.41)** |
|  |  |  | cRept |  |  | **-0.41 (-0.45 to -0.37)** |  |  |  | -0.41 (-1.05 to 0.22) |
|  | BM + taxon + trophic | 544 | a | -138.7 | 1.8 | **2.11 (2.07 to 2.15)** | **0.91 (0.85 to 0.94)** | -412.0 | 3.4 | **2.03 (1.51 to 2.55)** |
|  |  |  | b |  |  | **0.43 (0.42 to 0.45)** |  |  |  | **0.39 (0.37 to 0.42)** |
|  |  |  | cRept |  |  | **-0.40 (-0.45 to -0.35)** |  |  |  | -0.41 (-1.05 to 0.23) |
|  |  |  | dHerb |  |  | **0.06 (0.02 to 0.11)** |  |  |  | 0.02 (-0.05 to 0.09) |
|  |  |  | dOmni |  |  | -0.02 (-0.07 to 0.03) |  |  |  | 0.03 (-0.03 to 0.09) |
|  |  |  |  |  |  |  |  |  |  |  |
| Large intest. | BM | 528 | a | 462.9 | 429.4 | **1.61 (1.58 to 1.65)** | **0.95 (0.93 to 0.97)** | -218.9 | 35.5 | **1.27 (0.83 to 1.71)** |
|  |  |  | b |  |  | **0.49 (0.47 to 0.51)** |  |  |  | **0.38 (0.35 to 0.41)** |
|  | BM + trophic | 528 | a | 135.3 | 101.8 | **1.22 (1.18 to 1.27)** | **0.93 (0.88 to 0.96)** | -254.4 | 0.0 | **1.17 (0.79 to 1.55)** |
|  |  |  | b |  |  | **0.41 (0.39 to 0.43)** |  |  |  | **0.37 (0.34 to 0.40)** |
|  |  |  | dHerb |  |  | **0.65 (0.59 to 0.71)** |  |  |  | **0.27 (0.18 to 0.35)** |
|  |  |  | dOmni |  |  | **0.35 (0.28 to 0.41)** |  |  |  | **0.13 (0.06 to 0.20)** |
|  | BM + taxon | 528 | a | 275.4 | 241.9 | **1.75 (1.72 to 1.78)** | **0.95 (0.92 to 0.97)** | -217.9 | 36.5 | **1.57 (0.83 to 2.32)** |
|  |  |  | b |  |  | **0.41 (0.39 to 0.44)** |  |  |  | **0.38 (0.35 to 0.41)** |
|  |  |  | cRept |  |  | **-0.52 (-0.59 to -0.45)** |  |  |  | -0.47 (-1.39 to 0.46) |
|  | BM + taxon + trophic | 528 | a | 33.5 | 0.0 | **1.41 (1.36 to 1.46)** | **0.93 (0.88 to 0.96)** | -253.7 | 0.7 | **1.47 (0.83 to 2.11)** |
|  |  |  | b |  |  | **0.38 (0.36 to 0.39)** |  |  |  | **0.37 (0.34 to 0.39)** |
|  |  |  | cRept |  |  | **-0.33 (-0.39 to -0.27)** |  |  |  | -0.45 (-1.24 to 0.34) |
|  |  |  | dHerb |  |  | **0.52 (0.46 to 0.58)** |  |  |  | **0.27 (0.19 to 0.35)** |
|  |  |  | dOmni |  |  | **0.20 (0.14 to 0.26)** |  |  |  | **0.13 (0.06 to 0.20)** |
|  |  |  |  |  |  |  |  |  |  |  |
| Caecum | BM | 404 | a | 436.7 | 298.9 | **0.88 (0.84 to 0.92)** | **0.98 (0.97 to 0.99)** | -117.0 | 15.2 | 0.54 (-0.08 to 1.16) |
|  |  |  | b |  |  | **0.30 (0.27 to 0.33)** |  |  |  | **0.30 (0.26 to 0.33)** |
|  | BM + trophic | 404 | a | 222.1 | 84.3 | **0.35 (0.28 to 0.42)** | **0.97 (0.95 to 0.98)** | -132.2 | 0.0 | 0.39 (-0.19 to 0.96) |
|  |  |  | b |  |  | **0.24 (0.22 to 0.27)** |  |  |  | **0.29 (0.25 to 0.32)** |
|  |  |  | dHerb |  |  | **0.74 (0.65 to 0.82)** |  |  |  | **0.29 (0.17 to 0.42)** |
|  |  |  | dOmni |  |  | **0.51 (0.42 to 0.60)** |  |  |  | **0.20 (0.09 to 0.32)** |
|  | BM + taxon | 404 | a | 296.6 | 158.8 | **0.98 (0.94 to 1.01)** | **0.98 (0.97 to 0.99)** | -115.1 | 17.1 | 0.71 (-0.28 to 1.70) |
|  |  |  | b |  |  | **0.23 (0.21 to 0.26)** |  |  |  | **0.30 (0.26 to 0.33)** |
|  |  |  | cRept |  |  | **-0.72 (-0.83 to -0.61)** |  |  |  | -0.28 (-1.55 to 0.99) |
|  | BM + taxon + trophic | 404 | a | 137.8 | 0.0 | **0.56 (0.49 to 0.64)** | **0.97 (0.95 to 0.98)** | -130.5 | 1.7 | 0.58 (-0.33 to 1.50) |
|  |  |  | b |  |  | **0.20 (0.18 to 0.22)** |  |  |  | **0.29 (0.25 to 0.32)** |
|  |  |  | cRept |  |  | **-0.50 (-0.60 to -0.40)** |  |  |  | -0.32 (-1.49 to 0.85) |
|  |  |  | dHerb |  |  | **0.57 (0.49 to 0.66)** |  |  |  | **0.29 (0.17 to 0.42)** |
|  |  |  | dOmni |  |  | **0.29 (0.20 to 0.38)** |  |  |  | **0.21 (0.09 to 0.32)** |

**S6 Table** Summary statistics for models assessing different reptilian intestinal sections only with snout-vent-length (SVL) or additionally with whether the species is a snake or not, and /or a diet proxy according to log(length) = a + b log(SVL) + c (snake) + d (trophic level) , or y = (10^a^) (10^c^) (10^d^) SVL^b^; (significant parameters in **bold**)

| **Dependent** | **Model** |  |  | **GLS** |  | **parameter** | **PGLS** |  |  | **parameter** |
| --- | --- | --- | --- | --- | --- | --- | --- | --- | --- | --- |
|  |  | **n** |  | **AICc** | **ΔAIC** | **(95% CI)** | **lambda** | **AICc** | **ΔAIC** | **(95% CI)** |
| Total intest. | SVL | 118 | a | 15.9 | 65.0 | **0.22 (0.06 to 0.38)** | **0.91 (0.76 to 0.99)** | -101.3 | 18.0 | 0.16 (-0.11 to 0.43) |
|  |  |  | b |  |  | **0.98 (0.85 to 1.11)** |  |  |  | **1.02 (0.90 to 1.14)** |
|  | SVL + trophic | 118 | a | -22.9 | 26.2 | 0.13 (-0.01 to 0.26) | **0.85 (0.61 to 0.96)** | -114.2 | 5.2 | 0.22 (-0.02 to 0.46) |
|  |  |  | b |  |  | **0.93 (0.83 to 1.04)** |  |  |  | **0.94 (0.82 to 1.06)** |
|  |  |  | dHerb |  |  | **0.39 (0.29 to 0.50)** |  |  |  | **0.20 (0.11 to 0.29)** |
|  |  |  | dOmni |  |  | **0.22 (0.14 to 0.30)** |  |  |  | **0.08 (0.02 to 0.14)** |
|  | SVL + snake | 118 | a | -29.4 | 19.7 | -0.12 (-0.28 to 0.03) | **0.86 (0.63 to 0.97)** | -108.5 | 10.8 | 0.10 (-0.15 to 0.35) |
|  |  |  | b |  |  | **1.31 (1.17 to 1.44)** |  |  |  | **1.09 (0.96 to 1.22)** |
|  |  |  | cSnake |  |  | **-0.83 (-1.04 to -0.62)** |  |  |  | **-0.49 (-0.80 to -0.19)** |
|  | SVL + snake + trophic | 118 | a | -49.1 | 0.0 | -0.10 (-0.24 to 0.04) | **0.78 (0.45 to 0.94)** | -119.3 | 0.0 | 0.16 (-0.07 to 0.38) |
|  |  |  | b |  |  | **1.18 (1.06 to 1.31)** |  |  |  | **1.01 (0.88 to 1.14)** |
|  |  |  | cSnake |  |  | **-0.59 (-0.79 to -0.40)** |  |  |  | **-0.38 (-0.65 to -0.11)** |
|  |  |  | dHerb |  |  | **0.28 (0.18 to 0.38)** |  |  |  | **0.18 (0.09 to 0.28)** |
|  |  |  | dOmni |  |  | **0.16 (0.09 to 0.23)** |  |  |  | **0.08 (0.02 to 0.14)** |
|  |  |  |  |  |  |  |  |  |  |  |
| Small intest. | SVL | 116 | a | 22.6 | 42.7 | 0.14 (-0.02 to 0.29) | **0.73 (0.52 to 0.87)** | -67.6 | 6.4 | 0.19 (-0.06 to 0.45) |
|  |  |  | b |  |  | **0.94 (0.81 to 1.06)** |  |  |  | **0.92 (0.79 to 1.06)** |
|  | SVL + trophic | 116 | a | -2.2 | 17.9 | -0.03 (-0.17 to 0.12) | **0.70 (0.46 to 0.86)** | -72.4 | 1.6 | 0.17 (-0.08 to 0.42) |
|  |  |  | b |  |  | **0.95 (0.84 to 1.05)** |  |  |  | **0.90 (0.76 to 1.03)** |
|  |  |  | dHerb |  |  | **0.27 (0.15 to 0.38)** |  |  |  | **0.13 (0.02 to 0.24)** |
|  |  |  | dOmni |  |  | **0.27 (0.18 to 0.35)** |  |  |  | **0.11 (0.03 to 0.19)** |
|  | SVL + snake | 116 | a | -13.5 | 6.6 | **-0.24 (-0.41 to -0.07)** | **0.66 (0.39 to 0.84)** | -71.1 | 2.9 | 0.10 (-0.14 to 0.35) |
|  |  |  | b |  |  | **1.29 (1.15 to 1.43)** |  |  |  | **1.01 (0.87 to 1.16)** |
|  |  |  | cSnake |  |  | **-0.71 (-0.91 to -0.51)** |  |  |  | **-0.36 (-0.65 to -0.07)** |
|  | SVL + snake + trophic | 116 | a | -20.1 | 0.0 | **-0.27 (-0.43 to -0.10)** | **0.64 (0.35 to 0.83)** | -74.0 | 0.0 | 0.09 (-0.16 to 0.33) |
|  |  |  | b |  |  | **1.22 (1.07 to 1.36)** |  |  |  | **0.98 (0.83 to 1.13)** |
|  |  |  | cSnake |  |  | **-0.53 (-0.74 to -0.32)** |  |  |  | -0.29 (-0.58 to 0.00) |
|  |  |  | dHerb |  |  | **0.14 (0.02 to 0.26)** |  |  |  | 0.10 (-0.01 to 0.22) |
|  |  |  | dOmni |  |  | **0.19 (0.11 to 0.28)** |  |  |  | **0.10 (0.02 to 0.18)** |
|  |  |  |  |  |  |  |  |  |  |  |
| Large intest. | SVL | 111 | a | 100.2 | 50.9 | **-0.38 (-0.62 to -0.14)** | **0.95 (0.85 to 0.99)** | -2.6 | 13.3 | **-0.72 (-1.16 to -0.29)** |
|  |  |  | b |  |  | **0.98 (0.79 to 1.18)** |  |  |  | **1.14 (0.95 to 1.33)** |
|  | SVL + trophic | 111 | a | 55.5 | 6.2 | **-0.45 (-0.65 to -0.26)** | **0.90 (0.74 to 0.98)** | -12.5 | 3.4 | **-0.59 (-0.98 to -0.19)** |
|  |  |  | b |  |  | **0.87 (0.72 to 1.02)** |  |  |  | **1.00 (0.81 to 1.19)** |
|  |  |  | dHerb |  |  | **0.67 (0.51 to 0.83)** |  |  |  | **0.28 (0.14 to 0.42)** |
|  |  |  | dOmni |  |  | **0.23 (0.12 to 0.35)** |  |  |  | 0.06 (-0.03 to 0.16) |
|  | SVL + snake | 111 | a | 77.9 | 28.6 | **-0.78 (-1.04 to -0.53)** | **0.92 (0.80 to 0.99)** | -8.3 | 7.6 | **-0.82 (-1.24 to -0.40)** |
|  |  |  | b |  |  | **1.36 (1.14 to 1.58)** |  |  |  | **1.25 (1.05 to 1.45)** |
|  |  |  | cSnake |  |  | **-1.01 (-1.38 to -0.64)** |  |  |  | **-0.75 (-1.27 to -0.23)** |
|  | SVL + snake + trophic | 111 | a | 49.3 | 0.0 | **-0.65 (-0.88 to -0.43)** | **0.88 (0.71 to 0.97)** | -15.9 | 0.0 | **-0.68 (-1.07 to -0.29)** |
|  |  |  | b |  |  | **1.09 (1.89 to 1.29)** |  |  |  | **1.11 (0.90 to 1.32)** |
|  |  |  | cSnake |  |  | **-0.56 (-0.90 to -0.22)** |  |  |  | **-0.57 (-1.06 to -0.09)** |
|  |  |  | dHerb |  |  | **0.57 (0.41 to 0.73)** |  |  |  | **0.25 (0.10 to 0.39)** |
|  |  |  | dOmni |  |  | **0.17 (0.06 to 0.29)** |  |  |  | 0.05 (-0.05 to 0.15) |
|  |  |  |  |  |  |  |  |  |  |  |
| Caecum | SVL | 23 | a | 21.5 | 7.5 | **-0.88 (-1.23 to -0.53)** | 0.00 (NA to 0.64) | 13.7 | 8.8 | **-0.88 (-1.23 to -0.53)** |
|  |  |  | b |  |  | **0.75 (0.48 to 1.02)** |  |  |  | **0.75 (0.48 to 1.02)** |
|  | SVL + trophic | 23 | a | 23.1 | 9.1 | **-0.99 (-1.32 to -0.66)** | 0.00 (NA to 0.54) | 10.5 | 5.6 | **-0.99 (-1.32 to -0.66)** |
|  |  |  | b |  |  | **0.73 (0.48 to 0.97)** |  |  |  | **0.73 (0.48 to 0.97)** |
|  |  |  | dHerb |  |  | **0.35 (0.06 to 0.64)** |  |  |  | **0.35 (0.06 to 0.64)** |
|  |  |  | dOmni |  |  | 0.26 (-0.03 to 0.55) |  |  |  | 0.26 (-0.03 to 0.55) |
|  | SVL + snake | 23 | a | 14.0 | 0.0 | **-1.51 (-1.97 to -1.05)** | 0.00 (NA to 0.38) | 4.9 | 0.0 | **-1.51 (-1.97 to -1.05)** |
|  |  |  | b |  |  | **1.35 (0.94 to 1.75)** |  |  |  | **1.35 (0.94 to 1.75)** |
|  |  |  | cSnake |  |  | **-1.20 (-1.88 to -0.52)** |  |  |  | **-1.20 (-1.88 to -0.52)** |
|  | SVL + snake + trophic | 23 | a | 20.7 | 6.7 | **-1.43 (-1.93 to -0.92)** | 0.00 (NA to 0.39) | 7.3 | 2.4 | **-1.43 (-1.93 to -0.92)** |
|  |  |  | b |  |  | **1.21 (0.71 to 1.70)** |  |  |  | **1.21 (0.71 to 1.70)** |
|  |  |  | cSnake |  |  | **-0.93 (-1.79 to -0.07)** |  |  |  | **-0.93 (-1.79 to -0.07)** |
|  |  |  | dHerb |  |  | 0.15 (-0.17 to 0.48) |  |  |  | 0.15 (-0.17 to 0.48) |
|  |  |  | dOmni |  |  | 0.15 (-0.24 to 0.43) |  |  |  | 0.15 (-0.14 to 0.43) |

NA no model output

**S7 Table** Summary statistics for models assessing different lizard or turtle intestinal sections only with snout-vent-length (SVL) or additionally with a diet proxy according to log(length) = a + b log(SVL) + c (trophic level) , or y = (10^a^) (10^c^) SVL^b^; (significant parameters in **bold**)

| **Dependent** | **Model** |  |  | **GLS** |  | **parameter** | **PGLS** |  |  | **parameter** |
| --- | --- | --- | --- | --- | --- | --- | --- | --- | --- | --- |
|  |  | **n** |  | **AICc** | **ΔAIC** | **(95% CI)** | **lambda** | **AICc** | **ΔAIC** | **(95% CI)** |
| *Lizards & turtles (without snakes)* | | | | | | | | | | |
| Total intest. | SVL | 112 | a | -36.8 | 18.1 | **-0.19 (-0.34 to -0.03)** | **0.93 (0.74 to NA)** | -114.6 | 9.2 | **0.04 (-0.22 to 0.31)** |
|  |  |  | b |  |  | **1.36 (1.23 to 1.50)** |  |  |  | **1.14 (1.01 to 1.27)** |
|  | SVL + trophic | 112 | a | -54.9 | 0.0 | **-0.15 (-0.29 to -0.01)** | **0.88 (0.60 to 0.99)** | -123.8 | 0.0 | 0.10 (-0.14 to 0.35) |
|  |  |  | b |  |  | **1.22 (1.10 to 1.36)** |  |  |  | **1.05 (0.92 to 1.19)** |
|  |  |  | cHerb |  |  | **0.27 (0.17 to 0.36)** |  |  |  | **0.16 (0.08 to 0.25)** |
|  |  |  | cOmni |  |  | **0.16 (0.09 to 0.23)** |  |  |  | **0.07 (0.01 to 0.13)** |
|  |  |  |  |  |  |  |  |  |  |  |
| Small intest. | SVL | 107 | a | -23.9 | 7.3 | **-0.28 (-0.45 to -0.11)** | **0.86 (0.63 to 0.973)** | -90.4 | 2.7 | 0.02 (-0.25 to 0.29) |
|  |  |  | b |  |  | **1.32 (1.18 to 1.47)** |  |  |  | **1.08 (0.94 to 1.22)** |
|  | SVL + trophic | 107 | a | -31.2 | 0.0 | **-0.30 (-0.46 to -0.14)** | **0.84 (0.59 to 0.96)** | -93.1 | 0.0 | 0.01 (-0.25 to 0.28) |
|  |  |  | b |  |  | **1.25 (1.11 to 1.39)** |  |  |  | **1.05 (0.89 to 1.20)** |
|  |  |  | cHerb |  |  | **0.13 (0.02 to 0.24)** |  |  |  | 0.08 (-0.02 to 0.18) |
|  |  |  | cOmni |  |  | **0.19 (0.11 to 0.27)** |  |  |  | **0.09 (0.02 to 0.16)** |
|  |  |  |  |  |  |  |  |  |  |  |
| Large intest. | SVL | 106 | a | 71.2 | 27.2 | **-0.83 (-1.1 to -0.57)** | **0.94 (0.83 to 0.99)** | -16.1 | 6.9 | **-0.84 (-1.26 to -0.43)** |
|  |  |  | b |  |  | **1.40 (1.18 to 1.63)** |  |  |  | **1.27 (1.07 to 1.47)** |
|  | SVL + trophic | 106 | a | 44.0 | 0.0 | **-0.69 (-0.92 to -0.46)** | **0.92 (0.76 to 0.99)** | -23.0 | 0.0 | **-0.71 (-1.11 to -0.31)** |
|  |  |  | b |  |  | **1.13 (0.92 to 1.33)** |  |  |  | **1.13 (0.92 to 1.34)** |
|  |  |  | cHerb |  |  | **0.56 (0.40 to 0.72)** |  |  |  | **0.23 (0.09 to 0.37)** |
|  |  |  | cOmni |  |  | **0.17 (0.06 to 0.29)** |  |  |  | 0.05 (-0.05 to 0.14) |
|  |  |  |  |  |  |  |  |  |  |  |
| *Lizards* |  |  |  |  |  |  |  |  |  |  |
| Total intest. | SVL | 74 | a | -41.1 | 11.9 | -0.09 (-0.25 to 0.07) | **0.89 (0.61 to NA)** | -79.2 | 9.9 | -0.19 (-0.41 to 0.03) |
|  |  |  | b |  |  | **1.20 (1.05 to 1.35)** |  |  |  | **1.22 (1.06 to 1.39)** |
|  | SVL + trophic | 74 | a | -53.0 | 0.0 | 0.04 (-0.11 to 0.19) | **0.81 (0.38 to 0.99)** | -89.1 | 0.0 | -0.05 (-0.25 to 0.16) |
|  |  |  | b |  |  | **1.03 (0.89 to 1.18)** |  |  |  | **1.07 (0.91 to 1.24)** |
|  |  |  | cHerb |  |  | **0.30 (0.19 to 0.41)** |  |  |  | **0.23 (0.11 to 0.35)** |
|  |  |  | cOmni |  |  | 0.05 (-0.03 to 0.12) |  |  |  | 0.04 (-0.03 to 0.10) |
|  |  |  |  |  |  |  |  |  |  |  |
| Small intest. | SVL | 63 | a | -42.4 | 7.1 | **-0.22 (-0.37 to -0.07)** | **0.71 (0.35 to 0.93)** | -72.9 | 4.2 | **-0.33 (-0.52 to -0.13)** |
|  |  |  | b |  |  | **1.19 (1.05 to 1.33)** |  |  |  | **1.24 (1.09 to 1.40)** |
|  | SVL + trophic | 63 | a | -49.5 | 0.0 | -0.12 (-0.26 to 0.03) | **0.53 (0.10 to 0.86)** | -77.1 | 0.0 | **-0.19 (-0.37 to 0.00)** |
|  |  |  | b |  |  | **1.03 (0.90 to 1.17)** |  |  |  | **1.09 (0.94 to 1.25)** |
|  |  |  | cHerb |  |  | **0.27 (0.16 to 0.38)** |  |  |  | **0.19 (0.07 to 0.31)** |
|  |  |  | cOmni |  |  | 0.05 (-0.02 to 0.13) |  |  |  | 0.04 (-0.03 to 0.10) |
|  |  |  |  |  |  |  |  |  |  |  |
| Large intest. | SVL | 63 | a | -21.8 | 10.2 | **-0.88 (-1.05 to -0.70)** | **0.67 (0.25 to 0.91)** | -44.4 | 7.8 | **-0.91 (-1.15 to -0.67)** |
|  |  |  | b |  |  | **1.40 (1.23 to 1.56)** |  |  |  | **1.39 (1.20 to 1.57)** |
|  | SVL + trophic | 63 | a | -32.0 | 0.0 | **-0.73 (-0.89 to -0.56)** | 0.27 (NA to 0.85) | -52.2 | 0.0 | **-0.74 (-0.93 to -0.54)** |
|  |  |  | b |  |  | **1.20 (1.04 to 1.36)** |  |  |  | **1.20 (1.02 to 1.38)** |
|  |  |  | cHerb |  |  | **0.34 (0.21 to 0.46)** |  |  |  | **0.29 (0.14 to 0.43)** |
|  |  |  | cOmni |  |  | 0.04 (-0.05 to 0.12) |  |  |  | 0.03 (-0.06 to 0.12) |
|  |  |  |  |  |  |  |  |  |  |  |

***S7 Table ctd.*** Summary statistics for models assessing different lizard or turtle intestinal sections only with snout-vent-length (SVL) or additionally with a diet proxy according to log(length) = a + b log(SVL) + c (trophic level) , or y = (10^a^) (10^c^) SVL^b^; (significant parameters in **bold**)

| **Dependent** | **Model** |  |  | **GLS** |  | **parameter** | **PGLS** |  |  | **parameter** |
| --- | --- | --- | --- | --- | --- | --- | --- | --- | --- | --- |
|  |  | **n** |  | **AICc** | **ΔAIC** | **(95% CI)** | **lambda** | **AICc** | **ΔAIC** | **(95% CI)** |
| *Turtles* |  |  |  |  |  |  |  |  |  |  |
| Total intest. | SVL | 38 | a | -25.9 | 0.0 | **0.55 (0.20 to 0.90)** | **0.92 (0.00 to NA)** | -39.6 | 0.2 | **0.40 (0.08 to 0.72)** |
|  |  |  | b |  |  | **0.90 (0.63 to 1.16)** |  |  |  | **0.97 (0.77 to 1.17)** |
|  | SVL + trophic | 38 | a | -22.6 | 3.3 | 0.32 (-0.06 to 0.69) | 0.85 (NA to NA) | -39.8 | 0.0 | 0.29 (-0.07 to 0.64) |
|  |  |  | b |  |  | **0.97 (0.71 to 1.22)** |  |  |  | **0.98 (0.76 to 1.20)** |
|  |  |  | cHerb |  |  | **0.21 (0.07 to 0.35)** |  |  |  | 0.16 (-0.01 to 0.33) |
|  |  |  | cOmni |  |  | **0.16 (0.03 to 0.28)** |  |  |  | 0.14 (0.00 to 0.27) |
|  |  |  |  |  |  |  |  |  |  |  |
| Small intest. | SVL | 44 | a | -15.5 | 0.0 | **0.55 (0.16 to 0.94)** | **0.98 (0.56 to NA)** | -34.8 | 0.9 | **0.57 (0.22 to 0.92)** |
|  |  |  | b |  |  | **0.78 (0.47 to 1.08)** |  |  |  | **0.79 (0.58 to 1.00)** |
|  | SVL + trophic | 44 | a | -11.2 | 4.3 | 0.38 (-0.07 to 0.83) | **1.00 (0.59 to NA)** | -35.7 | 0.0 | 0.37 (-0.01 to 0.76) |
|  |  |  | b |  |  | **0.88 (0.57 to 1.18)** |  |  |  | **0.87 (0.66 to 1.08)** |
|  |  |  | cHerb |  |  | -0.09 (-0.26 to 0.09) |  |  |  | 0.05 (-0.14 to 0.23) |
|  |  |  | cOmni |  |  | 0.08 (-0.07 to 0.24) |  |  |  | 0.13 (-0.02 to 0.29) |
|  |  |  |  |  |  |  |  |  |  |  |
| Large intest. | SVL | 43 | a | 58.3 | 11.9 | 0.00 (-0.97 to 0.96) | **1.00 (0.80 to NA)** | 12.2 | 0.0 | **-0.67 (-1.29 to -0.06)** |
|  |  |  | b |  |  | **0.82 (0.08 to 1.56)** |  |  |  | **1.10 (0.75 to 1.46)** |
|  | SVL + trophic | 43 | a | 46.4 | 0.0 | -0.46 (-1.39 to 0.48) | **0.98 (0.70 to NA)** | 13.8 | 1.6 | -0.64 (-1.33 to 0.05) |
|  |  |  | b |  |  | **0.85 (0.22 to 1.48)** |  |  |  | **1.04 (0.65 to 1.43)** |
|  |  |  | cHerb |  |  | **0.87 (0.51 to 1.24)** |  |  |  | 0.22 (-0.11 to 0.55) |
|  |  |  | cOmni |  |  | **0.36 (0.04 to 0.69)** |  |  |  | 0.08 (-0.20 to 0.36) |

NA no model output

**S8 Table** Summary statistics for models assessing different reptilian intestinal sections only with body mass (BM) or snout-vent-length (SVL), or additionally with whether the species is a snake or not, and /or a diet proxy according to log(length) = a + b log(x) + c (snake) + d (trophic level) , or y = (10^a^) (10^c^) (10^d^) x^b^; (significant parameters in **bold**)

| **Dependent** | **Model** |  |  | **GLS** |  | **parameter** | **PGLS** |  |  | **parameter** |
| --- | --- | --- | --- | --- | --- | --- | --- | --- | --- | --- |
|  |  | **n** |  | **AICc** | **ΔAIC** | **(95% CI)** | **lambda** | **AICc** | **ΔAIC** | **(95% CI)** |
| Total intest. | BM | 82 | a | -75.2 | 0.0 | **1.77 (1.72 to 1.82)** | **0.58 (0.11 to 0.86)** | -96.1 | 8.8 | **1.77 (1.63 to 1.91)** |
|  |  |  | b |  |  | **0.40 (0.36 to 0.43)** |  |  |  | **0.38 (0.34 to 0.42)** |
|  | SVL | 82 | a | -12.2 | 63.0 | **0.25 (0.11 to 0.39)** | **0.91 (0.73 to 0.99)** | -63.3 | 41.6 | 0.09 (-0.24 to 0.42) |
|  |  |  | b |  |  | **0.87 (0.75 to 0.98)** |  |  |  | **1.04 (0.89 to 1.19)** |
|  | BM + trophic | 82 | a | -72.1 | 3.1 | **1.73 (1.66 to 1.79)** | **0.55 (0.14 to 0.83)** | -103.2 | 1.7 | **1.74 (1.60 to 1.87)** |
|  |  |  | b |  |  | **0.38 (0.35 to 0.41)** |  |  |  | **0.36 (0.32 to 0.40)** |
|  |  |  | dHerb |  |  | **0.16 (0.06 to 0.25)** |  |  |  | **0.18 (0.07 to 0.28)** |
|  |  |  | dOmni |  |  | 0.01 (-0.06 to 0.07) |  |  |  | 0.01 (-0.05 to 0.07) |
|  | SVL + trophic | 82 | a | -36.8 | 38.4 | **0.23 (0.11 to 0.35)** | **0.81 (0.51 to 0.96)** | -78.5 | 26.4 | 0.22 (-0.05 to 0.48) |
|  |  |  | b |  |  | **0.82 (0.72 to 0.92)** |  |  |  | **0.91 (0.77 to 1.04)** |
|  |  |  | dHerb |  |  | **0.39 (0.28 to 0.51)** |  |  |  | **0.29 (0.17 to 0.41)** |
|  |  |  | dOmni |  |  | **0.09 (0.01 to 0.17)** |  |  |  | 0.06 (-0.01 to 0.13) |
|  | BM + taxon | 82 | a | -70.6 | 4.6 | **1.76 (1.70 to 1.82)** | **0.54 (0.09 to 0.85)** | -95.1 | 9.8 | **1.76 (1.63 to 1.90)** |
|  |  |  | b |  |  | **0.39 (0.36 to 0.42)** |  |  |  | **0.38 (0.34 to 0.42)** |
|  |  |  | cSnake |  |  | 0.07 (-0.06 to 0.19) |  |  |  | 0.09 (-0.09 to 0.28) |
|  | SVL + taxon | 82 | a | -36.5 | 38.7 | -0.04 (-0.19 to 0.11) | **0.84 (0.55 to 0.97)** | -71.3 | 33.6 | 0.00 (-0.30 to 0.29) |
|  |  |  | b |  |  | **1.17 (1.03 to 1.31)** |  |  |  | **1.14 (0.99 to 1.30)** |
|  |  |  | cSnake |  |  | **-0.62 (-0.82 to -0.41)** |  |  |  | **-0.54 (-0.86 to -0.22)** |
|  | BM + taxon + trophic | 82 | a | -71.3 | 3.9 | **1.68 (1.61 to 1.75)** | **0.44 (0.06 to 0.78)** | -104.9 | 0.0 | **1.70 (1.58 to 1.82)** |
|  |  |  | b |  |  | **0.36 (0.32 to 0.39)** |  |  |  | **0.35 (0.31 to 0.38)** |
|  |  |  | cSnake |  |  | **0.14 (0.02 to 0.26)** |  |  |  | 0.16 (0.00 to 0.32) |
|  |  |  | dHerb |  |  | **0.19 (0.09 to 0.29)** |  |  |  | **0.20 (0.10 to 0.30)** |
|  |  |  | dOmni |  |  | 0.03 (-0.04 to 0.09) |  |  |  | 0.02 (-0.04 to 0.08) |
|  | SVL + taxon + trophic | 82 | a | -47.5 | 27.7 | 0.04 (-0.10 to 0.18) | **0.74 (0.32 to 0.93)** | -83.5 | 21.4 | 0.12 (-0.13 to 0.37) |
|  |  |  | b |  |  | **1.03 (0.90 to 1.16)** |  |  |  | **1.01 (0.86 to 1.16)** |
|  |  |  | cSnake |  |  | **-0.41 (-0.61 to -0.22)** |  |  |  | **-0.38 (-0.65 to -0.10)** |
|  |  |  | dHerb |  |  | **0.30 (0.19 to 0.41)** |  |  |  | **0.26 (0.14 to 0.38)** |
|  |  |  | dOmni |  |  | 0.07 (-0.01 to 0.14) |  |  |  | 0.05 (-0.02 to 0.11) |
|  |  |  |  |  |  |  |  |  |  |  |
| SVL | BM | 82 | a | -31.2 | 109.8 | **1.61 (1.54 to 1.68)** | **1.00 (0.99 to NA)** | -173.7 | 38.1 | **1.50 (1.33 to 1.66)** |
|  |  |  | b |  |  | **0.36 (0.31 to 0.40)** |  |  |  | **0.32 (0.29 to 0.35)** |
|  | BM + trophic | 82 | a | -33.2 | 107.8 | **1.70 (1.62 to 1.78)** | **1.00 (0.99 to NA)** | -171.8 | 40.0 | **1.51 (1.34 to 1.68)** |
|  |  |  | b |  |  | **0.37 (0.33 to 0.41)** |  |  |  | **0.33 (0.30 to 0.36)** |
|  |  |  | dHerb |  |  | **-0.19 (-0.31 to -0.07)** |  |  |  | -0.03 (-0.10 to 0.04) |
|  |  |  | dOmni |  |  | **-0.13 (-0.21 to -0.05)** |  |  |  | -0.02 (-0.06 to 0.01) |
|  | BM + taxon | 82 | a | -141.0 | 0.0 | **1.47 (1.43 to 1.51)** | **1.00 (0.98 to NA)** | -211.8 | 0.0 | **1.45 (1.32 to 1.58)** |
|  |  |  | b |  |  | **0.29 (0.27 to 0.31)** |  |  |  | **0.31 (0.29 to 0.33)** |
|  |  |  | cSnake |  |  | **0.65 (0.57 to 0.73)** |  |  |  | **0.59 (0.42 to 0.75)** |
|  | BM + taxon + trophic | 82 | a | -130.7 | 10.3 | **1.50 (1.45 to 1.54)** | **1.00 (0.98 to NA)** | -208.9 | 2.9 | **1.46 (1.33 to 1.60)** |
|  |  |  | b |  |  | **0.29 (0.27 to 0.32)** |  |  |  | **0.31 (0.29 to 0.33)** |
|  |  |  | cSnake |  |  | **0.63 (0.54 to 0.71)** |  |  |  | **0.58 (0.41 to 0.74)** |
|  |  |  | dHerb |  |  | -0.02 (-0.09 to 0.04) |  |  |  | -0.01 (-0.07 to 0.05) |
|  |  |  | dOmni |  |  | **-0.05 (-0.09 to 0.00)** |  |  |  | -0.01 (-0.04 to 0.01) |

NA no model output

|  |  |
| --- | --- |
|  |  |

**S1 Figure** Relationship of body mass and intestinal length in reptiles for (A) total intestine (n=157 species), (B) small intestine (n=147), (C) large intestine (Caecum and colon) (n=141), (D) caecum (n=52), as compared to mammals (light grey symbols). For statistics, see Table 1 for the reptile scaling, Table S1 for the scaling in individual reptile groups, and Table S2 for the comparison of reptiles and mammals. Note the generally lower values and, for (A) and (B), the less steep scaling in reptiles, and for (D) the steeper scaling in reptiles as compared to mammals. Mammal data from [3].
