# Supplementary material for: Less need for differentiation? Intestinal length of reptiles as compared to mammals
Source: PLoS One. 2021 Jul 2;16(7):e0253182. doi: 10.1371/journal.pone.0253182 (PMC8253402; doi:10.1371/journal.pone.0253182)
Supplement: S6 File — Reference list. (DOCX) [file pone.0253182.s006.docx]

**Less need for differentiation? Intestinal morphology of reptiles as compared to mammals**

**Monika I. Hoppe, Carlo Meloro, Mark S. Edwards, Daryl Codron, Marcus Clauss and María J. Duque-Correa**

**References for the datasets provided as excel files**

**References - digestive tract anatomy**

Barboza P (1995) Digesta passage and functional anatomy of the digestive tract in the desert tortoise *Xerobates agassizii*. Journal of Comparative Physiology B 165:193-202

Bauer M (2002) Untersuchungen zur vergleichenden Morphologie des Gastrointestinaltraktes der Schildkröten, Dissertation Thesis, Ludwig-Maximilian University Munich, Munich

Billing H (1987) Vergleichende anatomische und histologische Untersuchungen zur adaptiven Radiation des Verdauungstraktes bei Schlangen, Dissertation Thesis, Universitiy of Zurich, Zurich

Bjorndal K (1979) Cellulose digestion and volatile fatty acid production in the green turtle, *Chelonia mydas*. Comparative Biochemistry and Physiology A 63:127-133

Bjorndal K, Bolten A (1990) Digestive processing in a herbivorous freshwater turtle: consequences of small-intestine fermentation. Physiological Zoology 63:1232-1247

Christel C, DeNardo D, Secor S (2007) Metabolic and digestive response to food ingestion in a binge-feeding lizard, the Gila monster (*Heloderma suspectum*). Journal of Experimental Biology 210:3430-3439

Colferai A, Silva-Filho R, Martins A, Bugoni L (2017) Distribution pattern of anthropogenic marine debris along the gastrointestinal tract of green turtles (*Chelonia mydas*) as implications for rehabilitation. Marine Pollution Bulletin 119:231-237

De Queiroz M, Magalhães M, Vogt R (2009) Estudo morfológico do trato digestório de quatro espécies de quelônios da familia podocnemididae. Jornada de Iniciação Científica 18:408-410

Dunlap C (1955) Notes on the visceral anatomy of the gian leatherback turtle *(Dermochelys coriacea)*. The Bulletin of the Thulane Medical Faculty 14: 55-69

El-Bakry A, Abdeen A, Abo-Eleneen R (2012) Anatomical and morphometrical study of the alimentary canal of the lizard *Scincus scincus* and the snake *Natrix tessellata*. Life Science Journal 4:1010-1022

Hailey A (1997) Digestive efficiency and gut morphology of omnivorous and herbivorous African tortoises. Canadian Journal of Zoology 75:787-794

Herrel A, Vanhooydonck B, Van Damme R (2004) Omnivory in lacertid lizards: adaptive evolution or constraint? Journal of Evolutionary Biology 17:974-984

Jackson K, Perry G (2000) Changes in intestinal morphology following feeding in the brown treesnake, *Boiga irregularis*. Journal of Herpetology 34:459-462

Jegede H, Sonfada M, Salami S (2015) Anatomical studies of the gastrointestinal tract of the striped sand snake (*Psammophis sibilans*). Nigerian Veterinary Journal 36:1288-1298

Karasov W, Solberg D, Diamond J (1985) What transport adaptations enable mammals to absorb sugars and amino acids faster than reptiles? American Journal of Physiology-Gastrointestinal and Liver Physiology 249:G271-G283

Kopsch G (2006) Untersuchungen zur Körperzusammensetzung von Schildkröten, Ludwig-Maximillian Universtität Munich, Munich

Luz V, Stringhini J, de Bataus Y, de Paula W, Novais M, dos Reis I (2003) Morfometria do trato digestório da tartaruga-da-Amazônia (*Podocnemis expansa*) criada em sistema comercial. Revista Brasileira de Zootecnia 32:10-18

Lönnberg E (1902) On some points of the relation between the morphological structure of the intestine and the diet of reptiles. Kungliga Svenska Vetenskapsakademiens Handlingar 28:1-53

Mackie R, Rycyk M, Ruemmler R, Aminov R, Wikelski M (2004) Biochemical and microbiological evidence for fermentative digestion in free-living land iguanas (*Conolophus pallidus*) and marine iguanas (*Amblyrhynchus cristatus*) on the Galapagos archipelago. Physiological and Biochemical Zoology 77:127-138

Magalhães M, Vogt R, Barcellos J, Moura C, Da Silveira R (2014) Morphology of the digestive tube of the *Podocnemididae* in the Brazilian Amazon. Herpetologica 70:449-463

Mathes K, Radelof K, Engelke E, Rohn K, Pfarrer C, Fehr M (2019) Specific anatomy and radiographic illustration of the digestive tract and transit time of two orally administered contrast media in inland bearded dragons (*Pogona vitticeps*). PloS One 14:1-40

Ming-bin Y, Zhong-rong X, Hua-ling C, He-xiang GU, Jin-xia D, Fei-yan Z, Pei-peng L (2012) Anatomy of several systems in Olive Ridley sea turtle. Journal of Snake 24:237-240

Naya D, Veloso C, Bozinovic F (2008) Physiological flexibility in the Andean lizard *Liolaemus bellii*: seasonal changes in energy acquisition, storage and expenditure. Journal of Comparative Physiology B 178:1007-1015

Naya D, Veloso C, Sabat P, Bozinovic F (2009) Seasonal flexibility of organ mass and intestinal function for the Andean lizard *Liolaemus nigroviridis*. Journal of Experimental Zoology A 311:270-277

Naya D, Veloso C, Sabat P, Bozinovic F (2010) Seasonal flexibility in organ size in the Andean lizard *Liolaemus moradoensis*. Journal of Morphology 271:1440-1445

Ott B, Secor S (2007) Adaptive regulation of digestive performance in the genus python. Journal of Experimental Biology 210:340-356

Przystalski A (1980) The dimensions of the mucosa and the structure of the alimentary canal in some reptiles. Acta Biologica Cracoviensia 22:1-33

Rahman M, Sharma D (2014) Morphometric, anatomical and histological features of gastrointestinal tract (GIT) of freshwater turtle, *Pangshura tentoria*. International Journal of Scientific and Engineering Research 7:90-94

Rodrigues-Sartori S, Nogueira K, dos Santos Rocha A, Neves C (2014) Functional morphology of the gut of the tropical house gecko *Hemidactylus mabouia* (Squamata: Gekkonidae). Animal Biology 64:217-237

Santos X, Llorente G (2008) Gastrointestinal responses to feeding in a frequently feeding colubrid snake (*Natrix maura*). Comparative Biochemistry and Physiology A 150:75-79

Schneemeier C (2008) Untersuchung zur Körperzusammensetzung von Echsen, Dissertation Thesis, Ludwig-Maximillian Universität Munich, Munich

Slay C (2015) Plasticity along the cardiac-gastrointestinal axis in carnivorous reptiles, PhD Thesis, University of California, Irvine

Srichairat N, Taksintun W, Chumnanpuen P (2018) Gross morphological structure of digestive system in water monitor lizard *Varanus salvator* (Squamata: Varanidae). Walailak Journal of Science and Technology 15:245-253

Tracy C, Diamond J (2005) Regulation of gut function varies with life-history traits in chuckwallas (*Sauromalus obesus*: Iguanidae). Physiological and Biochemical Zoology 78:469-481

Tracy C, McWorther TJ, Gienger CM, Starck JM, Medley P, Manolis SC, Medley PS, Manolis G, Webb GJW, Kieth AC (2015) Alligators and crocodiles have high paracellular absorption of nutrients, but differ in digestive morphology and physiology. Integrative and Comparative Biology 55:986-1004

Valente AL, Marco I, Parga ML, Lavin S, Alegre F, Cuenca R (2008) Ingesta passage and gastric emptying times in loggerhead sea turtles *(Caretta caretta)*. Research in Veterinary Science 84: 132-139

Valido A, Nogales M (2003) Digestive ecology of two omnivorous canarian lizard species (*Gallotia*, Lacertidae). Amphibia-Reptilia 24:331-344

Vidal M, Sabat P (2010) Stable isotopes document mainland–island divergence in resource use without concomitant physiological changes in the lizard *Liolaemus pictus*. Comparative Biochemistry and Physiology B 156:61-67

**References – other biological characteristics**

Akani G, Luiselli L, Eniang E, Amuzie C, Ebere N (2007) Aspects of the ecology of the spotted blindsnake, *Typhlops punctatus punctatus* in Port-Harcourt, Nigeria. African Journal of Ecology 46: 533-539

Amr Z, Disi A (1997) Diet of some snakes from Jordan. Amphibia-Reptilia 19: 436-439

Andriantsaralaza S, Pedrono M, Tassin J, Roger E, Rakouth B, Danthu P (2013) The role of extinct giant tortoises in the germination of extant baobab Adansonia rubrostipa seeds in Madagascar. African Journal of Ecology 52: 246-249

Artner H (1995) Haltung und Nachtzucht von (*Chelodina reimanni)*, 1990 – mit Beobachtungen zu ihrem Lebensraum in Irian Jaya, Neuguinea. Herpetozoa 8: 17-24

Balensiefer DC and Vogt RC (2006). Diet of *Podocnemis unifilis* during the dry season in Mamirauá sustainable development reserve, Amazonas, Brazil. Chelonian Conservation and Biology 5: 312-317

Bamford M, Prendergast A (2017) A futher record and observations on the flowerpot snake *Indotyphlops braminus* in suburban Perth. The Western Australian Naturalist 30: 229-234

Barboza P (1995) Digesta passage and functional anatomy of the digestive tract in the desert tortoise *Xerobates agassizii*. Journal of Comparative Physiology B 165: 193-202.

Barlow A, Pook CE, Harrison RA, Wuster W (2009) Coevolution of diet and prey-specific venom activity supports the role of selection in snake venom evolution. Proceedings of the Royal Society B 276: 2443-2449

Beavers R (1976) Food habits of the western diamondback rattlesnake, *Crotalus atrox*, in Texas. The Southwestern Naturalist 20: 503-515

Bell I (2012) Algivory in hawksbill turtles: Eretmochelys imbricata food selection within a foraging area on the northern great barrier reef. Marine Ecology 34: 43-55

Bessesen BL, Galbreath GJ (2017) A new subspecies of sea snake, *Hydrophis platurus xantho*s, from Golfo Dulce, Costa Rica. Zookeys 686: 109-123

Bjorndal K (1979) Cellulose digestion and volatile fatty acid production in the green turtle, (*Chelonia mydas)*. Comparative Biochemistry and Physiology A 63: 127-133.

Bjorndal K, Bolten A (1990) Digestive processing in a herbivorous freshwater turtle: consequences of small-intestine fermentation. Physiological Zoology 63: 1232-1247

Brasil MA, de Freitas Horta G, Fraxe Neto JH, Barros TO, Rinaldi Colli G (2011) Feeding ecology of (*Acanthochelys spixii)* (Testudines, Chelidae) in the Cerrado of central Brazil. Chelonian Conservation and Biology 10: 91–101.

Camera BF, Miranda EBP, Ribeiro RP, Barros M, Draque J, Waller T, Micucci PA, Dambros CS, Strüssmann C (2019) Historical Assumptions about the predation patterns of yellow Anacondas (*Eunectes notaeus*): Are they infrequent feeders? Journal of Herpetology 53: 47-52

Canova L, Gentilli A (2008) Diet of the asp viper *Vipera aspis* in woodland habitats of the Po plain. Acta Herpetologica 3: 175-178

Claude J, Pritchard PC, Tong H, Paradis E, & Auffray JC (2004) Ecological correlates and evolutionary divergence in the skull of turtles: a geometric morphometric assessment. Systematic biology 53: 933-948.

Coheen MA (2017) Yellow-footed Tortoise, *Chelonoidis denticulata* the brazilian giant tortoise. The Tortuga Gazette 53:1-3

Colman LP, Sampaio CLS, Weber MI, Comin de Castilhos J (2014) Diet of olive ridley sea turtles, *Lepidochelys olivacea*, in the waters of Sergipe, Brazil. Bio One 13: 266-271

Colston T, Costa G, Vitt L (2010) Snake diet and the deep history hypothesis. Biological Journal of the Linnean Society 101: 476-486

Cottone A, Bauer A (2009) Sexual size dimorphism, diet, and reproductive biology of the Afro-Asian Sand Snake, *Psammophis schokari* (Psammophiidae). Amphibia-Reptilia 30: 331-340

Cunningham PL, Simang A (2008) Ecology of the bushmanland tent tortoise (*Psammobates tentorius verroxii*) in Southern Namibia. Chelonian Conservation and Biology 7: 119-124

De Neira L,Fowler E, Johnson MK (1985) Diets of giant tortoises and feral burros on volcan Alcedo, Galapagos. The Journal of wildlife management 49:165-169.

Del Vecchio S, Burke RL, Rugiero L, Capula M, Luiselli L (2011) Seasonal changes in the diet of *Testudo hermanni hermanni* in central Italy. Herpetologica 67: 236-249

Duda PL, Gupta VK (1980) Courtship and mating behaviour of the Indian soft shell turtle, *Lissemys punctata punctata*. Proceedings of the Indian Academy of Sciences. 90: 453-361

El-Bakry AM, Abdenn AM, Abo Eleneen RE (2012) Anatomical and morphometrical study of the alimentary canal of the lizard *Scincus scincus* and the snake *Natrix tessellata*. Life Science Journal 4: 1010-1022

El Mouden EH, Slimani T, Ben Kaddour K, Lagarde F, Ouhammou A, Bonnet X (2006) *Testudo graeca graeca* feeding ecology in an arid and overgrazed zone in Morocco. Journal of Arid Environments 64: 422-435

Elsey RM (2006) Food habits of *Macrochelys temminckii* (Alligator snapping turtle) from Arkansas and Louisiana. Southeastern Naturalist 5: 443-452

Georges A, Alacs E, Pauza M, Kinginapi F, Ona A, Eisemberg C (2008) Freshwater turtles of the Kikori Drainage, Papua New Guinea, with special references to the pig-nosed turtle*, Carettochelys insculpta*. Wildlife Research 35: 700-711

Georges A, and Rod K (1989) Dry-season distribution and ecology of (*Carettochelys insculpta)* in Kakadu National Park, northern Australia. Australian Wildlife Research 16: 323-335.

Gibbs HL, Sanz L, Chiucchi JE, Farrell TM, Calvete JJ (2011) Proteomic analysis of ontogenetic and diet-related changes in venom composition of juvenile and adult dusky pigmy rattlesnakes (*Sistrurus miliarius barbouri)*. Journal of Proteomics 74: 2169-2179

Glaudas X, Kearney TC, Alexander GJ (2017) Museum specimens bias measures of snake diet: A case study using the ambush-foraging Puff Adder *(Bitis arietans)*. Herpetologica 73: 121-128

Goetz M (2007) Husbandry and breeding of the spiny turtle *Heosemys spinosa* at the Durrell Wildlife Conservation Trust. Radiata 16: 1-15

Greene H (1983) Dietary correlates of the origin and radiation of snakes. American Zoologist 23: 431-441

Haider MK (2015) Feeding behavior of freshwater turtle (Pangshura smithii) with respect to seasonal temperature variation. Department of Life Sciences, The Islamia University of Bahamalpur Pakistan, Master Thesis

Hailey A (1997) Digestive efficiency and gut morphology of omnivorous and herbivorous African tortoises. Canadian Journal of Zoology 75: 787-794

Heaslip SG, Iverson SJ, Bowen WD, James MC (2012) Correction: jellyfish support high energy intake of leatherback sea turtles (*Dermochelys coriacea*): video evidence from animal-borne cameras. PLOS ONE 7(6): e33259

Henderson R, Noeske-Hallin T, Ottenwalder J, Schwartz A (1987) On the diet of the boa *Epicrates striatus* on Hispaniola, with notes on *E. fordi* and *E. gracilis*. Amphibia-Reptilia 8: 251-258

Hossain L, Sarker SU (1995) Reproductive biology of indian roofed turtle, *Kachuga tecta*, in Bangladesh. Chelonian Conservation and Biology 1: 226-227

Heuring C, Barber D, Rains N, Erxleben D, Martin C, Williams D, McElroy EJ (2019) Genetics, morphology and diet of introduced populations of the ant-eating Texas Horned Lizard (*Phrynosoma cornutum*). Scientific Reports 9: 1-14

Hossain ML, Sarker S, & Sarker NJ (2010) Food habits and feeding behaviour of Bengal eyed turtle (Morenia petersi) in Bangladesh. Bangladesh Journal of Zoology, 35: 213-222.

Jackson K, Perry G (2000) Changes in intestinal morphology following feeding in the brown treesnake, (*Boiga irregularis)*. Journal of Herpetology 34: 459-462.

Jegede HO, Sonfada ML, Salami SO (2015) Anatomical studies of the gastrointestinal tract of the striped sand snake (*Psammophis sibilans*). Nigerian Veterinary Journal, 36: 1288-1298.

Joshua QI (2008) Seasonal effects on the feeding ecology and habitat of *Chersina angulata* in the South Western Cape. Doctoral Thesis, University of the Western Cape

Kavanagh BT, Kwiatkowski MA (2016) Sexual dimorphism, movement patterns, and diets of *Sternotherus carinatus* (Razorback musk turtle). Southeastern Naturalist 15: 117-133

Kimmel CE (1980) A diet and reproductive study for selected species of Malaysian turtles. Master Thesis, Eastern Illinois University

Lagarde F, Bonnet X, Corbin J, Henen B, Nagy K, Mardonov B, Naulleau G (2003) Foraging behaviour and diet of an ectothermic herbivore: *Testudo horsfieldi*. Ecography 26: 236-242

Lee HJ, Park DS (2010). Distribution, habitat characteristics, and diet of freshwater turtles in the surrounding area of the Seomjin River and Nam River in southern Korea. Journal of Ecology and Environment, 33: 237-244.

Lemell P, Weisgram J (1996). Feeding Patterns of *Pelusios Castaneus*. Netherlands Journal of Zoology 47:429-441

Lemell P, Lemell C, Snelderwaard P, Gumpenberger M, Wochesländer R, & Weisgram J (2002) Feeding patterns of *Chelus fimbriatus*. Journal of Experimental Biology, *205*: 1495-1506.

Lescano J, Bonino M, Leynaud G, Haro J (2009) Diet of *Hydromedusa tectifera* (in a mountain stream of Córdoba province, Argentina. Amphibia-Reptilia, 30: 545-554.

Leuteritz TE, Walker RC (2013) *Pyxis arachnoides*. Turtles on the brink in Madagascar, p.50. Chelonian Research Monographs. Massachusetts, United States.

Lewis T, Grant P, Henderson R, Figueroa A, Dunn M (2011) Ecological notes on the annulated treeboa (*Corallus annulatus*) from a Costa Rican lowland tropical wet forest. IRCF Reptiles and Amphibians 18: 202-207

Luiselli L, Filippi E, Capula M (2005). Geographic variation in diet composition of the grass snake (*Natrix natrix*) along the mainland and an island of Italy: the effects of habitat type and interference with potential competitors. The Herpetological Journal *1*5: 221-230.

Luison A, Redaelli S (2008) Freshwater turltes and terrapins: a complete guide. Testud Edizioni, Tipografía Moderna, Italy. ISBN: 978-88-95662-01-5

Magalhães MS, Vogt RC, Barcellos JFM, Moura CEB, De Silveira R (2014). Morphology of the digestive tube of the *Podocnemididae* in the Brazilian Amazon. Herpetologica 70: 449-463.

McCormack TEM, Dawson JE, Hendrie DB, Ewert MA, Iverson JB, Hatcher RE, Goode JM (2014) Mauremys annamensis Vietnamese pond turtle, Annam Pond Turtle, Rùa Trung Bộ. Chelonian Research Monographs, 5: 1-14.

Meiri S (2018) Traits of lizards of the world: Variation around a successful evolutionary design. Global Ecology and Biogeography 27: 1168-1172

Milton SJ (2017) Plants eaten and dispersed by adult leopard tortoises *Geochelone Pardalis* (Reptilia: Chelonii) in the southern Karoo. South African Journal of Zoology 27: 45-49

Morrison M, Butterfield B, Ross S, Collins C, Walde A, Gray J, Hauge J, Munscher E (2019) The diet of the eastern musk turtle (*Sternotherus odoratus*) as it pertains to invasive snail consumption in a freshwater spring habitat in Texas. Herpetology Notes 12: 1133-1139

Mwaya RT, Malonza PK, Ngwava JM, Moll D, Schmidt FAC, Rhodin AGJ (2019) Malacochersus tornieri. The IUCN Red List of Threatened Species 2019: e.T12696A508210. https://dx.doi.org/10.2305/IUCN.UK.2019-1.RLTS.T12696A508210.en.

Nandu VS, Arora BM (2017) Study on the foraging behavior and diet preferences of indian star tortoises (*Geochelone elegans*) in Chinnar Wildlife Sanctuary Kerala, India. Journal of Zoological Sciences 5:58-69

Natusch DJD, Lyons JA (2014) Geographic and sexual variations in body size, morphology, and diet among five populations of Green Pythons (*Morelia viridis*). Journal of Herpetology 48: 317-323

Ott BD, Secor SM (2006) Adaptive regulation of digestive performance in the genus *Python*. Journal of Experimental Biology 210: 340-356

Paterson E (2018) The diet of African house snakes *(Boaedon spp)* revealed by citizen science. The Herpetological Bulletin 143: 29-30

Pellett S, Stocking D, Wissink-Argilaga N (2015) Tortoise feeding and nutritional requirements. Companion Animal, 20: 240-245.

Pérez-Emán JL, Paolillo A (1997) Diet of the pelomedusid turtle *Peltocephalus dumerilianus* in the Venezuelan Amazon. Journal of Herpetology*,* 31:173-179.

Petras D, Hempel B-F, Göçmen B, Karis M, Whiteley G, Wagstaff SC, Heiss P, Casewell NR, Nalbantsoy A, Süssmuth RD (2018) Community venomics reveals intra-species variations in venom composition of a local population of *Vipera kaznakovi* in Northeastern Turkey. BioRxiv

Platt SG, Hall C, Liu H, Borg CK (2009) Wet-season food habits and intersexual dietary overlap of Florida box turtles (*Terrapene carolina bauri*) on National Key Deer Wildlife Refuge, Florida. Southeastern Naturalist 8: 335-346

Plummer M (1981) Habitat utilization, diet and movements of a temperate arboreal snake (*Opheodrys aestivus)*. Journal of Herpetology 15: 425-432

Punzo F (1975) Studies on the feeding behavior, diet, nesting habits and temperature relationships of *Chelydra serpentina osceola*. Journal of Herpetology 9:207-210.

Rahman MS, Sharma DK (2014) Morphometric, anatomical and histological features of gastrointestinal tract of freshwater turtle, *Pangshura tentoria*. International Journal Scientific and Engineering Research 7: 90-94.

Rand S, Dugan B, Monteza H, Vianda D (1990) The diet of a generalized folivore: *Iguana iguana* in Panama. Journal of Herpetology 24: 211-214

Ray JM, Montgomery CE, Mahon HK, Savitzky AH, Lips KR (2012) Goo-Eaters: Diets of the neotropical snakes *Dipsas* and *Sibon* in central Panama. Copeia 2012: 197-202

Rifai L, Amr ZS (2006) Diet of the stripe-necked terrapin, (Mauremys rivulata), in Jordan. Russian Journal of Herpetology, 13: 41-46.

Riyanto A, Wirdateti W, Soemarno S (2019) Observations on natural foods and nutrition content of critically endangered turtle (Leucocephalon yuwonoi) in central Sulawesi. Biota, 11: 87-91.

Rodríguez-Cabrera T, Fong A, Torres A (2020) New dietary records for three Cuban snakes in the genus Tropidophis with comments on possible niche partitioning by Cuban tropes. IRCF Reptiles and Amphibians 27: 207-208

Rodríguez-Robles J, Bell C, Greene H (1999) Gape size and evolution of diet in snakes: feeding ecology of ercyne boas. Journal of Zoology 248: 49-58

Santos X, Llorente GA (2008) Gastrointestinal responses to feeding in a frequently feeding colubrid snake (*Natrix maura*). Comparative Biochemistry and Physiology Part A 150: 75-79.

Schneider L, Belger L, Burger J, Vogt RC (2009) Mercury bioacumulation in four tissues of *Podocnemis erythrocephala* as a function of water parameters. Science of the Total Environment 407: 1048-1054

Seney EE, Musick JA (2007) Historical diet and analaysis of loggerhead sea turtles (*Caretta caretta*) in Virginia. Copeia 2: 478-489

Sidis I, Gasith A (1985). Food habits of the Caspian terrapin (*Mauremys caspica rivulata*) in unpolluted and polluted habitats in Israel. Journal of Herpetology 19: 108-115.

Sivan J, Kam M, Hadad S, Degen AA, Rozenboim I, Rosenstrauch A (2013) Temporal activity and dietary selection in two coexisting desert snakes, the Saharan sand viper (*Cerastes vipera*) and the crowned leafnose (*Lytorhynchus diadema*). Zoology 116: 113-117

Shine R, Branch W, Harlow P, Webb J, Shine T (2006) Biology of burrowing asps (Atractaspididae) from Southern Africa. American Society of Ichthyologists and Herpetologists 2006: 103-115

Smales L, El-Emarah GY, Essa IaM, Abdulzahra HK, Al-Azizz SA (2020) First Records of *Oligacanthorhynchus* (Oligacanthorhynchidae) from the Honey Badger, *Mellivora capensis wilsoni* (Mustelidae) and the West-Asian Blunt-Nosed Viper *Macrovipera lebetina obtusa* (Viperiidae) from North Basrah, Iraq. Comparative Parasitology 87: 44-48

Smith TL, Povel GDE, Kenneth KV (2006) Predatory strike of the tentacled snake (*Erpeton tentaculatum*). Journal of Zoology 256: 233-242

Souza F, Abe A (2000) Feeding ecology, density and biomass of the freshwater turtle, *Phrynops geoffroanus*, inhabiting a polluted urban river in south-eastern Brazil. Journal of Zoology 252: 437-446

Stauffer KE (2003) Captive care of the African spurred tortoise, Geochelone sulcata. Journal of Herpetological Medicine and Surgery, 13: 38-44.

Sung Y, Hau BCH, Karraker NE (2016) Diet of the endangered big-headed turtle *Platysternon megacephalum*. PeerJ e2784

Tracy C, McWorther TJ, Gienger CM, Starck JM, Medley P, Manolis SC, Medley PS, Manolis G, Webb GJW, Kieth AC (2015) Alligators and crocodiles have high paracellular absorption of nutrients, but differ in digestive morphology and physiology. Integrative and Comparative Biology 55:986-1004

Van Dijk PP, Harding J, Hammerson GA (2011). Trachemys scripta (errata version published in 2016). The IUCN Red List of Threatened Species 2011: e. T22028A97429935

Wallach VS (2010) Report on bites by the West African Night Adder, *Causus caculatus*. The Journal of the Herpetological Association of Africa 22: 3-8

Walde A, Bider JR, Daigle C, Masse D, Bourgeois JC, Jutras J, Titman RD (2003) Ecological aspects of a wood turtle *Glyptemys insculpta*, population at the northern limit of its range in Québec. The Canadian Field-Naturalist 117: 377-388

Wanchai P, (2007). Radio-telemetry study of home range size and activities of the black Asian giant tortoise *Manouria emys* *phayrei.* Doctoral dissertation, Chulalongkorn University

Wang E, Donatti CI, Ferreira VL, Raizer J, Himmelstein J (2011) Food habits and notes on the biology of *Chelonoidis carbonaria* (Spix 1824) (Testudinidae, Chelonia) in the southern Pantanal, Brazil. South American Journal of Herpetology 6: 11-19.

Welsh MA, Doody JS, Georges A (2017) Resource partitioning among five sympatric species of freshwater turtles from the wet–dry tropics of northern Australia. Wildlife Research 44.3: 219-229.

Wilhelm CE, Plummer MV (2012) Diet of radiotracked musk turtles, *Sternotherus Odoratus*, ina small urban stream. Herpetological Conservation and Biology 7: 258-264

**References – body mass calculation from snout vent length / carapax length (incl. data used for own derivation of equation for Podocnemis spp.)**

Bernhard R, Vogt R (2012) Population structure of the turtle *Podocnemis erythrocephala in the rio Negro basin, Brazil*. Herpetologica 68: 491-504

Fachín-Terán A, Vogt R, Thorbjarnarson J (2004) Patterns of use and hunting of turtles in the Mamirauá sustainable development reserve, Amazonas, Brazil. In: Silvius K, Bodmer R, Fragoso J (eds) People in Nature: Wildlife Conservation in South and Central America. Columbia University Press, pp 362-377

Feldman A, Meiri S (2013) Length-mass allometry in snakes. Biological Journal of the Linnean Society 108: 161-172

Hirth H (1982) Weight and length relationships of some adult marine turtles. Bulletin of Marine Science 32: 336-341

Meiri S (2010) Length-weight allometries in lizards. Journal of Zoology 281: 218-226

Miorando P, Giarrizzo T, Pezzuti J (2015) Population structure and allometry of *Podocnemis unifilis* (Testudines, Podocnemididae) in a protected area upstream Belo Monte dam in Xingu river, Brazil. Annals of the Brazilian Academy of Sciences 87: 2067-2079

Oliveira-Júnior A, Tavares-Dias M, Marcon J (2009) Biochemical and hematological reference ranges for amazon fresh water turtle, *Podocnemis expansa* (Reptilia: Pelomedusidae), with morphologic assessment of blood cells. Research in Veterinary Science 86: 146-151

Rossini M, Blanco P, Marín E, Comerma-Steffensen S, Zerpa H (2012) Haematological values of post laying arrau turtle (*Podocnemis expansa*) in the Orinoco river, Venezuela. Research in Veterinary Science 92: 128-131

Sá V, Quintanilha L, Freneau G, Ferreira Luz V, de los Reyes Borja A, Silva P (2004) Body growth of one-month giant amazonian turtle (*Podocnemis expansa*) fed isocaloric diet with different levels of crude protein concentration. Revista Brasileira de Zootecnia 33: 2351-2358

Smith N (1979) Aquatic turtles of amazonia: an endangered resource. Biological Conservation 16: 165-176
