# Supplementary material for: Less need for differentiation? Intestinal length of reptiles as compared to mammals
Source: PLoS One. 2021 Jul 2;16(7):e0253182. doi: 10.1371/journal.pone.0253182 (PMC8253402; doi:10.1371/journal.pone.0253182)
Supplement: Attachment — Submitted filename: ReplyLetter_210519_mc.docx [file pone.0253182.s007.docx]

PONE-D-21-11487

Reply to Reviewers

Dear Ulrich Joger,

Thank you for giving us the opportunity to revise our manuscript and respond to the Reviewers' comments.

Please find attached the revised manuscript with all revised text in grey shading. Our response to specific editor and reviewer comments are given at the bottom of this letter.

We thank you for your time and hope the manuscript now meets the requirements of the PLOSOne.

Sincerely,

Marcus Clauss & Maria Duque

See detailed responses to reviewers down below

***************************************************************************

Editor: The reviewer I found for your manuscript found nothing “wrong” in this paper with methods or statistics, and the writing appeared clear.

Reviewer 1: This manuscript analyzes and compares in reptiles and terrestrial mammals variation in intestine length in relation to body size, diet, and phylogeny. The statistical analysis of the data seem fine, and the writing is clear ...

I reiterate that I found nothing “wrong” in this paper with methods or statistics, and the writing is clear.

Editor: Yet he proposed to reject it. I am not so hard and like to give you a chance to improve the paper.

***Response: Thank you. We think that this is an expressive strategy of PLoS One. The publication criteria do not list something like «novelty». Because our study produced several «negative» results, which many journals shun, we decided to submit to PLoS One.***

------------------------------------------------------------------------------------------------------------------------------------------------------

Editor: Please enlarge the data (if you have more)

***Response: We do not have additional data we could add (if we had, we would have added it right away). We want to state here that our dataset is – compared to previously published ones – really large (> 200 species vs. 40). The reviewer referred to data on intestine diameter, and intestinal surface – data that does not exist at this moment. As the reviewer observed ...***

Reviewer 1: The authors recognize this (i) by pointing out that most authors who consider herbivorous reptile gut ecomorphology focus on the caeco-colonic dilatation and valve-like structures (lines 369-371), and (ii) with statements that additional data on gut diameter, which are necessary to make stronger inferences related to gut length, are not available (line 299,314-320, 329).

***... we mentioned several times in the original manuscript that data availability regarding these measures is a limiting factor, making explanations that refer to these measures speculative. We now also added this even once more in the form of an outlook for future studies.***

***new text l. 302 in revised manuscript:***

This hypothesis would have to be tested in two ways: (i) with a large dataset on intestinal diameter; (ii) with a large dataset on intestinal surfaces, which would test the underlying assumption that this surface scales geometrically with body mass. Neither dataset exists or can be collated easily, to our knowledge, and thus resolution of these question hinges on the future measurement of anatomical data.

***new text l. 447 in revised manuscript:***

In particular, future studies could fruitfully combine a variety of measurements, not only intestinal lengths, but organ tissue and contents mass, diameters and surfaces, as well as both body mass and snout-vent-length or carapax length, to facilitate more comprehensive comparisons that have to rely less on inductive speculation.

------------------------------------------------------------------------------------------------------------------------------------------------------

Editor: or change your conclusions by carefully differentiating between hard evidence and speculation. It is not a shame if a hypothesis is not supported by a data set, but a discussion must be self-critical.

***Response: We were surprised by this statement. We suspect it is the consequence of this statement by ...***

Reviewer 1: I mainly fault it for its narrow focus that to my thinking leads to conclusions poorly supported by data

***... which we think is not correct. What the editor demands – a clear differentiation between hard evidence and speculation – had been part of the original manuscript all along. Whenever there was a speculation, a word like «possibly» was placed in the corresponding sentence.***

***For example, note this comment of ...***

Reviewer 1: many points of discussion seem thin and speculative. For example, this seems to be the case for every point discussed in the section “Comparison with mammals” (lines 306-358). Because the issue of differences in gut diameter is unresolved, the authors try to infer it using an inconclusive argument (lines 310-320) followed finally by invoking the idea that mammals do have smaller intestine diameters (lines 329-330), which they do not have strong evidence for.

***Response: (i) it is not our fault that the argument about gut diameter is inconclusive – this is what results if you use all available information from the literature, as we did in l. 310-320 of the original manuscript. The only way to avoid the impression of ‘inconclusive’ would have been to omit one of the conflicting literature findings, which would be bad scholarship. (ii) We later re-used one specific idea, but we did not ‘invoke the idea that mammals do have smaller diameters’ but had clearely labelled this as speculation by using the word ‘possibly’ in that sentence***

l. 329 of the original manuscript, maintained unchanged as l. 335 in the revised manuscript

[mammals] possibly have shorter diffusion distances in the intestines via smaller intestine diameters (as deducted from the similar gut capacity and the longer intestines demonstrated in the present study)

***Additional indicators of speculation had included using the indicator «possibly» in l. 331, 348, 404, 413; «possible reason» in l. 301; we had listed constraint of the study in l. 259-288; we called the diameter-length relationship a «hypothesis» in l. 299 and another suggestion «hypothesis» in l. 429; we stated that the discrepancy in the literature remains unresolved in l. 320; we indicated speculation verbatim by stating «we can only speculate...» in l. 357 and l. 395; we used the word «might» in l. 402 and the words «may well derive» in l. 410; and finally, we had concluded with the statement that «clearly, more data are required to fully address these questions» in l. 438.***

***Hence, we are unsure how to further correspond to the demand of clearly separating speculation from hard evidence – we think we did that very clearly. To even further avoid the impression that we make unjust assertions, we added another caveat in the first paragraph of the discussion, l. 257 of the revised version:***

Even though our data collection represents possibly the largest one available to date, presented also in the hope that it will be useful for other researchers, the limitations of both the dataset itself and the focus on a single kind of measurement – length measures – must be stated.

------------------------------------------------------------------------------------------------------------------------------------------------------

Reviewer 1: I mainly fault it for its narrow focus that to my thinking leads to conclusions poorly supported by data, and little advance in more reliable understanding, as opposed to speculation.

***Response: Our response to this is twofold.***

***(i) Our study did produce hard results.***

***- as in mammals, the general allometric scaling of intestine length with body mass is higher than expected based on geometry; except for snakes***

***- reptiles have shorter intestines than mammals of similar body mass***

***- reptiles and mammals differ in the scaling exponent for the caecum***

***- in reptiles, there is only a weak trophic signal in intestine length – in contrast to mammals***

***- in reptiles, the intestinal section linked to the (weak) trophic signal is the large intestine***

***- the effect of body elongation on intestine length differs between snakes (reptiles) and mustelids (mammals)***

***It is the explanation of these results that must remain speculative, not the results themselves. As explained above, we always indicate the speculative part of the explanation.***

***(ii) Previously, similar or different conclusions have been based on distinctively smaller datasets. The finding that a larger dataset does not corroborate conclusions based on fewer data, but contradicts it, is also an important scholarly achievement.***

***The reviewer seems to ignore this, which also becomes evident in a specific comment made ...***

Reviewer 1: At lines 353-358 – I do not see why the authors state that liver size is probably similar in mammals and reptiles. The data from Else and Hulbert (1985) suggest otherwise, as do the regression lines shown in Fig. 1c of Franz et al. (2009).

***Response: Here, we have a similar situation. The dataset of Else and Hulbert (1985) was included in the larger dataset of Franz et al. (2009). The analysis of Franz et al. was not based on a visual impression of regression lines (which are indeed given as Fig. 1c in that paper) but on the presence or absence of 95% confidence interval overlap. In Franz et al. (2009), we had written “The 95% CI of the*** [reptile]  ***intercept of the ln-transformed equation (ln(a)) included values for birds and mammals in the case of the liver, indicating that, irrespective of the scaling pattern with BM, the actual mass of this organ is similar among the three vertebrate clades in the body size range studied”. So, we cited our 2009 paper correctly. Again, the 2009 paper is an example where a previous claim (by Else and Hulbert) was not corroborated by a larger dataset. This may be disappointing, but it is good scholarship to report this.***

***we added the note of the reviewer in the revised manuscript l. 358 to add the perspective***

Individual studies comparing organ masses between selected mammal and reptile species of similar body mass indicate larger organ masses in mammals (e.g. Else and Hulbert 1981, 1985). Larger literature compilations of data from several studies show that ...

------------------------------------------------------------------------------------------------------------------------------------------------------

Reviewer 1: The authors finish this section with the sentence “we can only speculate that reptiles may have distinctively more body tissue in the form of their tails”.

***Response: Again, this is proof that we indicate speculation; we are not sure what the criticism is at this point. However, we want to point out that to our knowledge, we are the first to ever raise this question. Several other authors have found before that reptiles have, at similar body mass, smaller organs than mammals. But the evident question arising from this – what is it that reptiles have to fill the difference (to same body mass) has never been raised before. We believe that raising this question is good scholarship. In preparing for the manuscript, one of us (MC) actually contacted several ‘reptile researchers’ to find out their opinion about this, and none of those contacted had been aware of the evident question, or knew a plausible answer.***

------------------------------------------------------------------------------------------------------------------------------------------------------

Reviewer 1: My main critique is that the paper advances knowledge about digestion or ecomorphology very little because it focuses on one thing, really one dimension, intestine length. As a reader, I approach it with about as much interest as I would a thorough analysis of some other intestinal analogue such as intestine width. Width could be analyzed in the same way as length is here, but who would be interested in that? Put the two together and one would certainly have a lot more, such as surface area that can be related to substrate absorption or niche space for adherent microbes, or gut volume that can be related to holding capacity for digesta or microbes. Add time (another dimension) and one can make inferences about turnover kinetics and the matching of rates such as digesta breakdown or metabolic demand. But working with gut length (or width) alone limits saying much definitive about function.

***Response:***

***(i) The objection that the focus of the manuscript is too narrow because it focusses on one measure only (length) (a) ignores the fact that we assess lengths of different intestinal sections, not just ‘one measure’, and (b) ignores the fact that compared to previous studies (with a maximum n = about 40), an n of about 200 is a really substantial increase. This critique would apply to all kinds of comparative studies (like all those comparative studies than only assess one measurement – basal metabolic rate). It is a major ‘problem’ or ‘characteristic’ of comparative studies that they focus on a certain measure and do not provide data on all the additional measures one would also like to have.***

***(ii) Our study aims at investigating length, and not give a review on all possible measures of digestive capacity. This is because length has a history – people have claimed that it is related to trophic niches. While this need not necessarily appeal to everyone, it should be relevant to all those who deal with reptile anatomy.***

***(iii) The reviewer states that she/he would like to see additional data on gut volume, digesta holding capacity, microbes, digesta turnover kinetics, metabolic demand, digesta breakdown. In our view, there is no way to link the data that exists for those aspects in a statistically meaningful way with the anatomical data – because hardly ever was data generated in the same specimens, and because using the data that exists will lead to much-reduced datasets compared to the one we built here for intestine lengths. However, in the discussion, we explicitly address all these issues, citing reviews on metabolism, digestion, microbiome, digesta holding capacity, retention times ... in doing this, we briefly do paint the comprehensive picture the reviewer wants. But in doing so, we also clearly outline the lacking knowledge, the lacking data – which shows that still, a lot remains speculative (which we mark as such). In our view, this is not a poor approach, but a comprehensive use of all existing knowledge.***

***in order to react to the statement, we***

***- added a sentence to the outlook section l. 447 of the revised manuscript***

In particular, future studies could fruitfully combine a variety of measurements, not only intestinal lengths, but organ tissue and contents mass, diameters and surfaces, as well as both body mass and snout-vent-length or carapax length, to facilitate more comprehensive comparisons that have to rely less on inductive speculation.

***- added a statement pointing out that we provide a huge data collection for the scientific community l. 257 of the revised manuscript***

Even though our data collection represents possibly the largest one available to date, presented also in the hope that it will be useful for other researchers, the limitations of both the dataset itself and the focus on a single kind of measurement – length measures – must be stated.

***- mention sample size more explicitly in l. 374***

Herrel et al. (2004) found that it was particularly the small intestine that was longer in four omnivorous vs. eleven faunivorous lizards

------------------------------------------------------------------------------------------------------------------------------------------------------

Reviewer 1: As another example, they speculate about differences in scaling exponent of intestine length (beginning line 338), which is perhaps not even meaningful without knowing intestine diameter and hence actual surface area. According to Karasov and Hume (1997) there is no difference in the scaling exponent of intestine nominal surface area between reptiles (5 species) and mammals (20 species). That’s not a lot of evidence either, so if the science needs more data then the best solution is to provide more data.

***Response: We do not speculate about differences in scaling exponents, we show them. We speculate about the meaning of the shown differences. We offer explanations that are plausible but require other data to test them – a common practice in the writing of discussion of scientific papers.***

***As mentioned before,***

***we added new text l. 302 in revised manuscript:***

This hypothesis would have to be tested in two ways: (i) with a large dataset on intestinal diameter; (ii) with a large dataset on intestinal surfaces, which would test the underlying assumption that this surface scales geometrically with body mass. Neither dataset exists or can be collated easily, to our knowledge, and thus resolution of these question hinges on the future measurement of anatomical data.

***and we added a sentence to the outlook section l. 447 of the revised manuscript***

In particular, future studies could fruitfully combine a variety of measurements, not only intestinal lengths, but organ tissue and contents mass, diameters and surfaces, as well as both body mass and snout-vent-length or carapax length, to facilitate more comprehensive comparisons that have to rely less on inductive speculation.

------------------------------------------------------------------------------------------------------------------------------------------------------

Reviewer 1: Testing putative trophic differences in intestine length was the “raison d’etre” for the paper (lines 26-28; 51-55). In the Discussion of this (lines 360-365) they point out that in mammals it applies mainly to the large intestine, which they also cite as Lonnberg’s conclusion for reptiles >100 years ago. It is what the authors found again here, mainly in lizards. The advance in knowledge that they offer is that the trophic signal might be weaker in reptiles than in mammals, though “larger datasets lead to stronger trophic signals” (lines 390-391). So, they end this section of the Discussion with speculation “…about reasons for a putatively less distinct effect of trophic level on intestine length in reptiles than in mammals” (395-396).

***Response: We explained in l. 62-63 of the original submission that the finding of Loennberg > 100 years ago was an observation that was not submitted to statistical testing. Claiming that the statistical corroboration of an old, un-tested observation does not represent progress may be up for debate, but it definitely is good scholarship. We test the observation using the original data only, and we additionally corroborate the observation with a larger dataset. We do not claim novelty or super-exciting results (although for some of us authors this is exciting stuff). And yes, the fact that trophic differentiation in intestine length is not as strong in reptiles as it is in mammals is what we offer as a new knowledge, as also reflected in our choice of title.***
